# Deciphering the interplay between psychopathological symptoms, sensorimotor, cognitive and global functioning: a transdiagnostic network analysis

**DOI:** 10.1007/s00406-024-01782-3

**Published:** 2024-03-20

**Authors:** Stefan Fritze, Geva A. Brandt, Sebastian Volkmer, Jonas Daub, Maria Krayem, Jacqueline Kukovic, Emanuel Schwarz, Urs Braun, Georg Northoff, Robert Christian Wolf, Katharina M. Kubera, Andreas Meyer-Lindenberg, Dusan Hirjak

**Affiliations:** 1 Department of Psychiatry and Psychotherapy, Central Institute of Mental Health, Medical Faculty Mannheim, University of Heidelberg, 68159 Mannheim, Germany; 2 Hector Institute for Artificial Intelligence in Psychiatry, Central Institute of Mental Health, Medical Faculty Mannheim, Heidelberg University, Mannheim, Germany; 3 Mind, Brain Imaging and Neuroethics Research Unit, The Royal’s Institute of Mental Health Research, University of Ottawa, Ottawa, ON Canada; 4 Center for Psychosocial Medicine, Department of General Psychiatry, University of Heidelberg, Heidelberg, Germany; 5 German Centre for Mental Health (DZPG), Partner Site Heidelberg/Mannheim/Ulm, Mannheim, Germany

**Keywords:** Psychopathology, Sensorimotor domain, Cognitive domain, Network analysis, Transdiagnostic

## Abstract

**Background:**

Understanding the relationship between psychopathology and major domains of human neurobehavioral functioning may identify new transdiagnostic treatment targets. However, studies examining the interrelationship between psychopathological symptoms, sensorimotor, cognitive, and global functioning in a transdiagnostic sample are lacking. We hypothesized a close relationship between sensorimotor and cognitive functioning in a transdiagnostic patient sample.

**Methods:**

We applied network analysis and community detection methods to examine the interplay and centrality [expected influence (EI) and strength] between psychopathological symptoms, sensorimotor, cognitive, and global functioning in a transdiagnostic sample consisting of 174 schizophrenia spectrum (SSD) and 38 mood disorder (MOD) patients. All patients (*n* = 212) were examined with the Positive and Negative Syndrome Scale (PANSS), the Heidelberg Neurological Soft Signs Scale (NSS), the Global Assessment of Functioning (GAF), and the Brief Cognitive Assessment Tool for Schizophrenia consisted of trail making test B (TMT-B), category fluency (CF) and digit symbol substitution test (DSST).

**Results:**

NSS showed closer connections with TMT-B, CF, and DSST than with GAF and PANSS. DSST, PANSS general, and NSS motor coordination scores showed the highest EI. Sensory integration, DSST, and CF showed the highest strength.

**Conclusions:**

The close connection between sensorimotor and cognitive impairment as well as the high centrality of sensorimotor symptoms suggests that both domains share aspects of SSD and MOD pathophysiology. But, because the majority of the study population was diagnosed with SSD, the question as to whether sensorimotor symptoms are really a transdiagnostic therapeutic target needs to be examined in future studies including more balanced diagnostic groups.

**Supplementary Information:**

The online version contains supplementary material available at 10.1007/s00406-024-01782-3.

## Introduction

The co-occurrence of psychopathological symptoms stemming from different functional domains is characteristic of psychiatric disorders. For instance, schizophrenia spectrum disorders (SSD) are characterized by psychopathological symptoms from various functional domains, including sensory–perceptual (e.g., hallucinations), cognitive (e.g., delusions, impaired working memory and executive functioning), affective (e.g., flat affect, anhedonia and depressive symptomatology [1]), somatic (e.g., fatigue, muscle pain, gastrointestinal symptoms and tension [2]), social (e.g., social isolation and autism), and sensorimotor (e.g., rigor, tremor, psychomotor slowing and akinesia [3]), respectively. In mood disorders (MOD), such as major depressive disorder (MDD) and bipolar disorder (BD), patients not only experience low mood, sadness [4], low self-esteem, lack of drive, and loss of interest or pleasure but also co-occurring cognitive changes (e.g., rumination [5] and impaired working memory [6]), somatic-neurovegetative (chest pain, gastrointestinal symptoms, irritable bowel, or other [7]), interpersonal (e.g., social anxiety and withdrawal [8]), sensory–perceptual (aberrant visual perception [9]), and motor (psychomotor retardation or agitation [10]) symptoms.

Furthermore, the majority of the psychopathological symptoms mentioned above are core diagnostic criteria for SSD and MOD by the most widely used diagnostic systems in clinical practice, such as the DSM-5 and ICD-11. The overlap and co-occurrence of psychopathological symptoms in SSD and MOD are specifically mentioned in both diagnostic systems as the foundation for differential diagnostic considerations. However, their intrinsic relationships—that is, in what ways do the various psychopathological symptoms interact and which symptoms are central or influential across SSD and MOD—remain understudied. In the last few years, novel statistical methods have been developed to explore the interplay between psychopathological symptoms and global functioning in psychiatric disorders [11–13]. In particular, network analysis can help identifying which psychopathological symptoms are central or influential within a network of different clinical variables [14, 15]. Central psychopathological symptoms are those that have strong connections to other symptoms or clinical variables and may play a crucial role in the development and maintenance of the disorder. Therefore, network analysis is a promising method to investigate the complexity of interactions between psychopathological symptoms stemming from different functional domains across different psychiatric disorders. Instead of seeing psychopathological symptoms as standalone entities, network analysis conceives of them as components of a complex network that interact with one another and frequently reinforce one another. In line with this, psychiatric disorders, such as SSD and MOD, might arise from the direct interactions of symptoms within a network architecture.

In the last two years, the network analysis method has contributed to a considerable increase in knowledge in the field of different psychopathological symptoms [16–18]. However, the studies using network analysis method have investigated mainly the interaction between different categories of psychopathological symptoms using single items stemming from different rating scales such as the Brief Negative Symptom Scale (BNSS) or the Positive and Negative Syndrome Scale (PANSS) in uni-diagnostic psychiatric samples [16–18].

Based on the recent evidence that distinct Neurological Soft Signs (NSS) have been shown to be differentially associated with psychopathology as well as cognition in SSD [19–21] and BD [22], the main goal of this study was to examine the associations among psychopathological symptoms, sensorimotor, cognitive, and global functioning in a transdiagnostic patient sample. We hypothesized that sensorimotor dysfunction, as examined with the Heidelberg NSS scale [23] would be more closely connected with cognition (as assessed with the Brief Cognitive Assessment Tool for Schizophrenia [B-CATS] [24]) than psychopathological symptoms examined with PANSS. Further, we hypothesized that sensorimotor dysfunction would be a central network component (as assessed with expected influence [EI] and strength). Unlike previous studies that conducted the network analyses at the single-item level, and because we had a strong prior theory on how variables are related, we decided to use subscale scores in order to examine associations between subdomains. If we confirmed our hypotheses, this study could point toward shared domain-based aspects of pathophysiology in SSD and MOD. This could point toward new treatment options for cognition, e.g., with stimulation techniques in specific brain regions associated with sensorimotor dysfunction [25, 26] or virtual reality [27].

## Methods

### Study participants

In this study, we combined two independent cohorts of patients from different studies conducted at the Central Institute of Mental Health (CIMH).

*Cohort #1* consisted of 129 subjects fulfilling the DSM-IV-TR [28] criteria for schizophrenia spectrum disorders (SSD) and 7 subjects fulfilling the DSM-IV-TR [28] criteria for bipolar disorders (BD) [29, 30]. This cohort has been used in previous studies of our group [3, 31, 32]. Diagnoses were made by staff psychiatrists and confirmed using the German versions of the Structured Clinical Interview for DSM-IV-TR axis I and II disorders (SCID) and examination of the case notes (SF and DH).

*Cohort #2* consisted of 76 subjects fulfilling the German Mini Diagnostic Interview for Mental Disorders (Mini-DIPS) [33] criteria for SSD (*n* = 45), major depressive disorder (MDD, *n* = 26) or BD (*n* = 5). Diagnoses were made by staff psychiatrists and confirmed using the examination of the case notes (SF, GAB and DH).

Patients in both cohorts were excluded if: (i) they were aged < 18 or > 65 years; (ii) they had a history of brain trauma or neurological disease (especially movement disorders); (iii) they had shown alcohol/substance use disorder within 12 months prior to participation.

The local Ethics Committees I and II (Medical Faculty Heidelberg and Medical Faculty Mannheim at Heidelberg University, Germany) approved the studies. We obtained written informed consent from all study participants after all aims and procedures of the study had been fully explained.

### Clinical assessment

Patients in both cohorts were examined during in- or outpatient treatment after partial remission of acute psychopathological symptoms. All relevant study procedures (e.g., psychopathological rating scales, neuropsychological assessments and sensorimotor assessment) were completed within 7 days. All SSD and MOD patients were on a stable daily dose of antipsychotic, antidepressant or mood-stabilizing medication for at least 7 days. Antipsychotic and antidepressant medication was standardized as Olanzapine [OLZe] [34] and fluoxetine [FLX] [35] equivalents (s. Table 1 for details). OLZe and FLXe were z-transformed, summed up and included as covariates (medication) in subsequent analyses (see below).

**Table 1:** Clinical and demographic variables of study participants (*n* = 212)

Variable	Mean ± SD	Skewness	Kurtosis
Age	37.88 ± 12.22	–	–
Sex (m/f)	104/108	–	–
Education (years)	13.28 ± 2.87	–	–
OLZe	14.39 ± 11.31	0.59	2.91
FLXe	7.84 ± 18.44	2.28	7.04
PANSS positive	14.32 ± 6.47	1.11	4.10
PANSS negative	16.87 ± 7.33	0.64	2.56
PANSS general	33.91 ± 9.91	0.93	4.68
PANSS total score	65.10 ± 19.74	0.66	3.30
NSS MOCO	7.07 ± 4.01	0.62	3.05
NSS SI	3.32 ± 1.79	0.71	4.26
NSS COMT	3.32 ± 2.26	0.63	3.00
NSS RLSPO	2.84 ± 2.39	1.51	6.75
NSS HS	3.23 ± 1.91	0.24	2.59
NSS total score	19.78 ± 8.63	0.88	3.41
GAF*	54.29 ± 16.84	0.07	2.35
CF*	35.41 ± 18.70	-0.02	1.62
DSST*	47.30 ± 22.22	0.11	2.28
TMT-B (seconds)	107.25 ± 61.48	1.62	5.61

Data are mean ± standard deviation

*SD* standard deviation, *m* male*, f* female*, OLZe* Olanzapine equivalents*, FLX* fluoxetine equivalents*, PANSS* positive and negative syndrome scale, *NSS* neurological soft signs, *MOCO* motor coordination, *SI* sensory integration, *RLSPO* right/left spatial orientation, *HS* hard signs, *CF* category fluency, *DSST* digit symbol substitution test, *TMT-B* trail making test part B

*GAF, CF, and DSST are reverse coded values

Patients were examined with the PANSS [36] for psychopathological assessment, including the following PANSS subscores: positive, negative and general. The Heidelberg NSS scale [23] including its sub-scores motor coordination (MOCO), sensory integration (SI), hard signs (HS), complex motor tasks (COMT), right/left spatial orientation (RLSPO) was used for sensorimotor assessment. General functioning was assessed with the Global Assessment of Functioning (GAF) scale [37]. Cognitive functioning was assessed with B-CATS [24], which consisted of trail making test B (TMT-B), category fluency (CF), and digit symbol substitution test (DSST). One of the main prerequisites for this study was to obtain the largest possible sample with overlapping psychopathological, sensorimotor, cognitive, and global assessments. Therefore, we opted for scales/assessments that are feasible and map the four domains we were interested in. Further, we decided on scales/assessments that have been previously used in the three disorders [38–40]. Therefore, PANSS, NSS, B-CATS, and GAF seemed to us to be the most suitable. Finally, we believe that the included scales PANSS, NSS, B-CATS, and GAF also allow good comparability with other studies.

### Statistical analyses

We used *R* version 4.0.4 and *Rstudio* version 1.3.1093 [41]. Initially, a descriptive analysis of demographic and clinical data was performed. Then, the homogeneity of variances and the normality of PANSS, NSS, GAF, and B-CATS were investigated using Levene’s test and Shapiro–Wilk. Further analyses were performed with PANSS sub-scores (positive, negative, and general), NSS sub-scores (MOCO, HS, COMT, RLSPO, SI), GAF score, and B-CATS sub-scores (TMT-B, DSST, CF). We used sub-scores in order to examine differential associations between subdomains rather than broad global scores which may mask associations. Since higher values of the variables GAF, CF, and DSST indicated better performance (while on the remaining scales, higher values indicated worse performance), GAF, CF, and DSST were converted reversely.

Using these scores, we have carried out the following statistical analyses: First, in line with previously published studies [14, 42–44], potentially relevant covariates (regression variables), such as age, sex, education, and medication, were not included in our analyses. Second, for completeness, we (A) regressed out age, sex, education, and medication and employed the residuals of PANSS sub-scores, NSS subscale scores, B-CATS sub-scores, and GAF score for network estimation and (B) included age, sex, education, and medication nodes in the network analyses (NA2-A and NA2-B) and EGA.

In the first and second step, we employed network analysis methods [45, 46]. In this approach, nodes represent variables, and lines between variables are termed edges, representing relationships to be investigated. The most common psychological network estimation technique uses partial correlations between variables (nodes), which are calculated after accounting for all other variable correlations. Blue edges refer to positive correlations, while red edges refer to negative correlations. Thicker edges refer to stronger correlations, while thinner edges refer to weaker correlations. Due to the violation of normality in our variables, we used a non-paranormal transformation in preparing the data. Employing the qgraph package, we set the default option of the estimateNetwork function to “huge”. Network estimation involved a graphical Gaussian model (GGM) [45, 47]. We applied the extended Bayesian information criterion (EBIC) [47, 48]. To avoid false-positive relationships between nodes, we regularized the network with the least absolute shrinkage and selection operator (LASSO) [47, 48]. LASSO can shrink small edges toward zero. Fruchterman–Reingold algorithm was used for visualization [48]. This algorithm places nodes with stronger correlations more centrally. To assess centrality, expected influence (EI) and strength were calculated. EI of a node represents the sum of all partial correlations (accounting for negative edges) and indicates its importance within a network graph. Higher values indicate stronger interconnectedness. Strength is the sum of absolute edge weights. To examine EI and strength stability, we employed a bootstrap approach with the bootnet package. Furthermore, and in order to investigate whether our results may have been driven by diagnosis, we repeated the network analyses including only SSD participants (*n* = 174, s. Supplementary Figs. 6–12). We refrained from repeating the analyses including only MOD patients (BD: *n* = 12; MDD: *n* = 24) or analyzing the two study cohorts (NSS cohort: *n* = 129; whiteCAT cohort: *n* = 83) separately, due to the small number of participants in terms of network analysis.

Then, in order to employ community detection methods on PANSS, NSS, GAF, and B-CATS, we used exploratory graph analysis (EGA) [49–51]. To this end, we employed the EGAnet package. EGA calculates polychoric correlations with the walktrap algorithm in order to identify communities of the partial correlation matrix [49]. The walktrap algorithm calculates similarities between vertices hinged on random walks [49]. Additionally, item stability was evaluated with 1000 bootstrapped samples, assessing the proportion of times specific items clustered with their community. Network loadings for each community were investigated with EGAnet. Loadings are interpreted as the node´s expected influence on each community. Higher loadings indicate that a node has a higher influence on the occurrence of a community.

Third, to validate the results of the network analyses, a partial correlation (C) was run to determine the relationship between PANSS positive, negative and general score, NSS subscale scores, B-CATS subscores, and GAF score while controlling for age, sex, education, and medication.

## Results

### Clinical and demographic characteristics

The study cohort (*n* = 212) consisted of participants with SSD (*n* = 174), MDD (*n* = 26) and BD (*n* = 12). Mean age and education were 37.88 years and 13.28 years, respectively. There were 104 male and 108 female participants. Detailed demographic and clinical characteristics of participants across all diagnoses and the included variables as well as skewness and kurtosis for each variable are shown in Table 1 (for group differences between variables see Supplementary Table 1).

### Network analysis

**Fig. 1:**
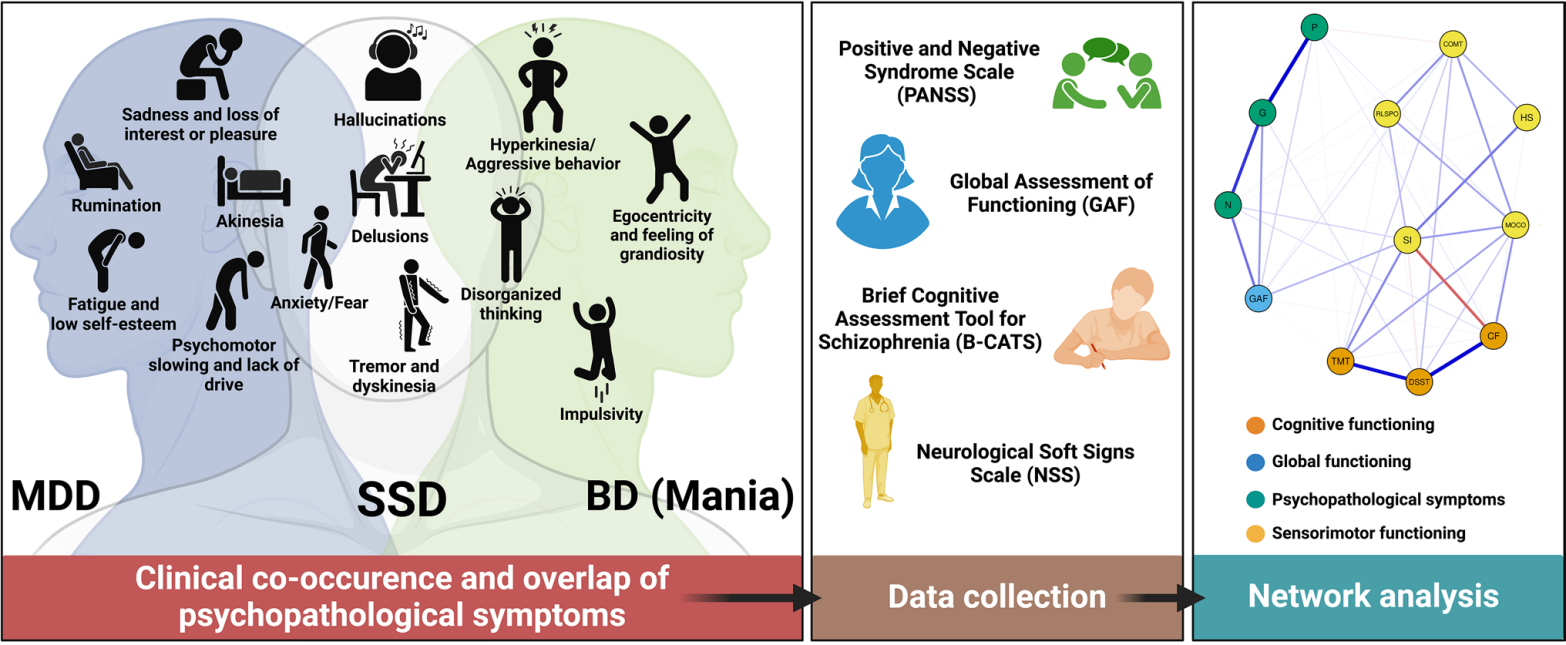
This figure describes the methodological approach of the study including the network structures of psychopathological symptoms, sensorimotor, cognitive, and global functioning in a transdiagnostic sample. Node colors in the estimated multi-dimensional network reflect the four functional domains. Blue edges represent positive associations; red edges represent negative associations. Thickness and saturation of edges indicate the strength of these associations

**Fig. 2:**
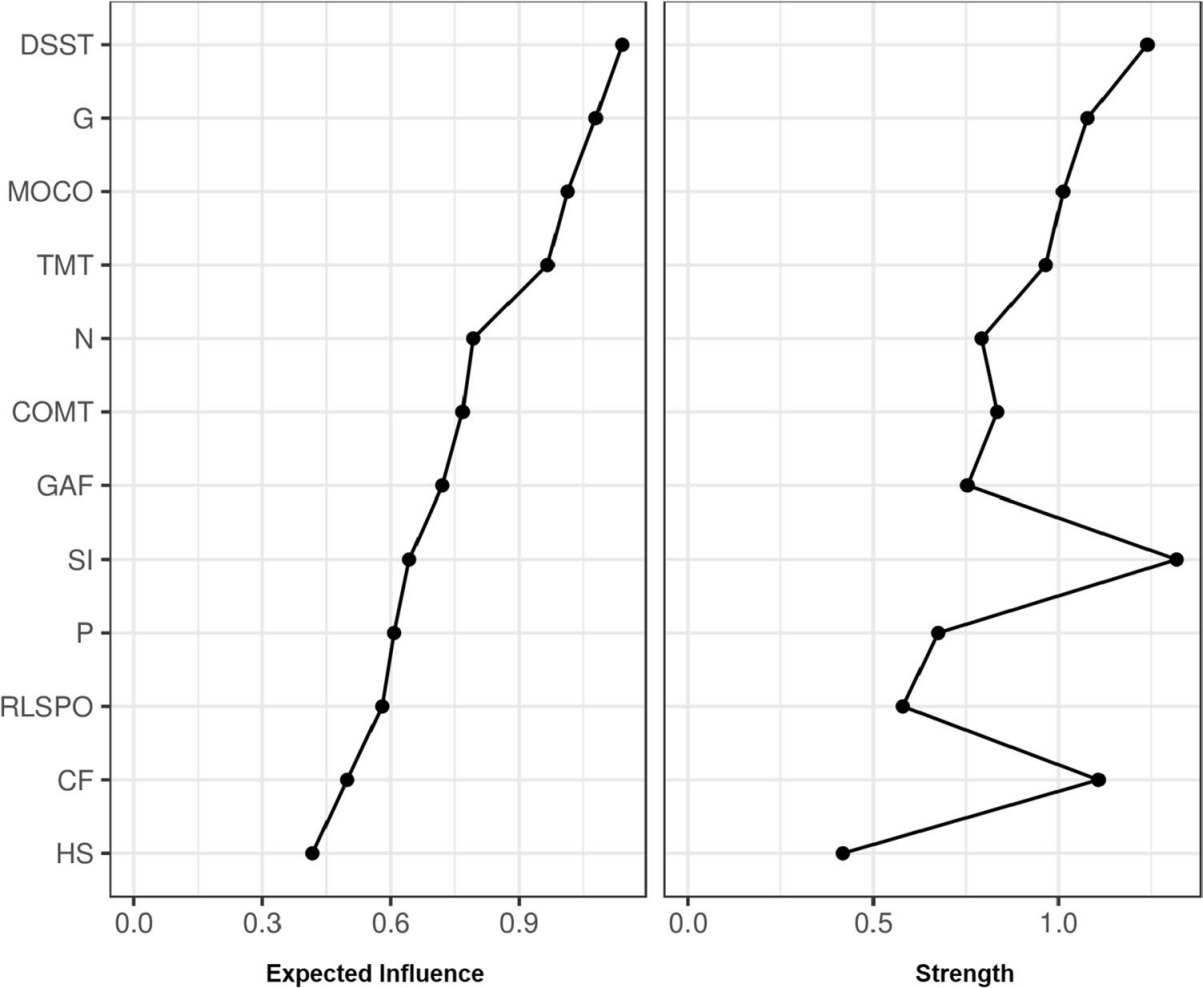
Centrality of the estimated multi-domain network. DSST: digit symbol substitution test; G: PANSS, general psychopathology dimension; MOCO: Neurological Soft Signs—Motor Coordination; TMT: Trail Making Test B; N: PANSS, negative symptoms; COMT: Neurological Soft Signs—Complex motor tasks; GAF: Global Assessment of Functioning; SI: Neurological Soft Signs—Sensory Integration subscale; P: PANSS, positive symptoms; RLSPO: Neurological Soft Signs—Right/Left and Spatial Orientation; CF: Category Fluency; HS: Neurological Soft Signs – Hard Signs

First, the network included the NSS, the PANSS, and the B-CATS sub-scores and GAF total scores (Fig. 1). Edges as well as expected influence (EI) and strength were deemed stable (s. Supplementary Figs. 1 and 2). The scores of the five NSS subscales showed low to no association with PANSS positive, negative, and general scores (Fig. 1, Table 2). MOCO showed associations with the three B-CATS sub-scores (Fig. 1, Table 2). SI showed an inverse association with CF and a positive association with TMT-B (Fig. 1, Table 2). The remaining NSS sub-scores showed fewer associations with B-CATS sub-scores (Fig. 1, Table 2). GAF was associated with PANSS scores and NSS IF sub-scores (Fig. 1, Table 2). DSST, PANSS general, MOCO, and TMT-B showed the largest EI as a measure of centrality (Fig. 2). EI of MOCO was not significantly different from DSST, PANSS general, or TMT-B EI (s. Supplementary Fig. 3), indicating similar centrality. Also, SI, DSST, and CF showed the highest strength as another measure of centrality, followed by PANSS general and MOCO (Fig. 2). SI strength was not significantly different from *strength* of either DSST, CF, PANSS general, MOCO, or TMT-B, indicating similar centrality (s. Supplementary Fig. 3).

**Table 2:** Partial correlation matrix

	PANSS positive	PANSS negative	PANSS general	NSS MOCO	NSS SI	NSS COMT	NSS RLSPO	NSS HS	GAF	CF	DSST	TMT-B
PANSS positive	0	0	0.470	0.032	0	− 0.034	0.010	0	0.080	0.038	0	0.012
PANSS negative	0	0	0.368	0	0.076	0.019	0.009	0.009	0.258	0.054	0	0
PANSS general	0.470	0.368	0	0	0	0	0	0	0.186	0	0.055	0
NSS MOCO	**0.032**	**0**	**0**	0	0.180	0.187	0.161	0	0	**0.193**	**0.097**	**0.163**
NSS SI	**0**	**0.076**	**0**	0.180	0	0	0.133	0.242	0.134	− **0.288**	− **0.050**	**0.216**
NSS COMT	− **0.034**	**0.019**	**0**	0.187	0	0	0.201	0.142	0.050	**0**	**0.117**	**0.086**
NSS RLSPO	**0.010**	**0.009**	**0**	0.161	0.133	0.201	0	0	0.022	**0.007**	**0.013**	**0.022**
NSS HS	**0**	**0.009**	**0**	0	0.242	0.142	0	0	0.004	**0.020**	**0**	**0**
GAF	0.080	0.258	0.186	0	0.135	0.050	0.022	0.004	0	− 0.017	0	0.003
CF	0.038	0.054	0	0.193	− 0.288	0	0.007	0.020	− 0.017	0	0.469	0.022
DSST	0	0	0.055	0.097	− 0.050	0.117	0.013	0	0	0.469	0	0.441
TMT-B	0.012	0	0	0.163	0.216	0.086	0.022	0	0.003	0.022	0.441	0

Partial correlations between variables in our study sample (*n* = 212). Higher values indicate stronger associations between variables. Connections between NSS (particularly MOCO and SI) are stronger with cognition than with psychopathology

*PANSS* positive and negative symptoms scale, *NSS* neurological soft signs, *MOCO* motor coordination, *SI* sensory integration, *RLSPO* right/left spatial orientation, *HS* hard signs, *CF* category fluency, *DSST* digit symbol substitution test, *TMT-B* trail making test part B

*GAF, CF, and DSST are reverse coded values

Second, the network (NA2-A) estimated after regressing out age, sex, education, and medication was unstable and thus could not be interpreted and the results are not shown. Unstable in this context refers to low probability of being able to reproduce the estimated network structure, including edges. Further, the network analysis (NA2-B) including age, sex, education, and medication as additional nodes was sufficiently stable (s. Supplementary Figs. 15 and 16) and the results remained comparable to our network analysis without these four variables (NA1): NSS still showed closer connections with TMT-B, CF and DSST than with GAF and PANSS (s. Supplementary Fig. 13 and supplementary Table 2). PANSS general, DSST, TMT, and NSS MOCO scores showed the highest EI, while DSST, SI, and TMT showed the highest strength (s. Supplementary Fig. 14). Overall, age, sex, education, and medication showed low centrality (s. Supplementary Fig. 14).

### Community detection

**Fig. 3:**
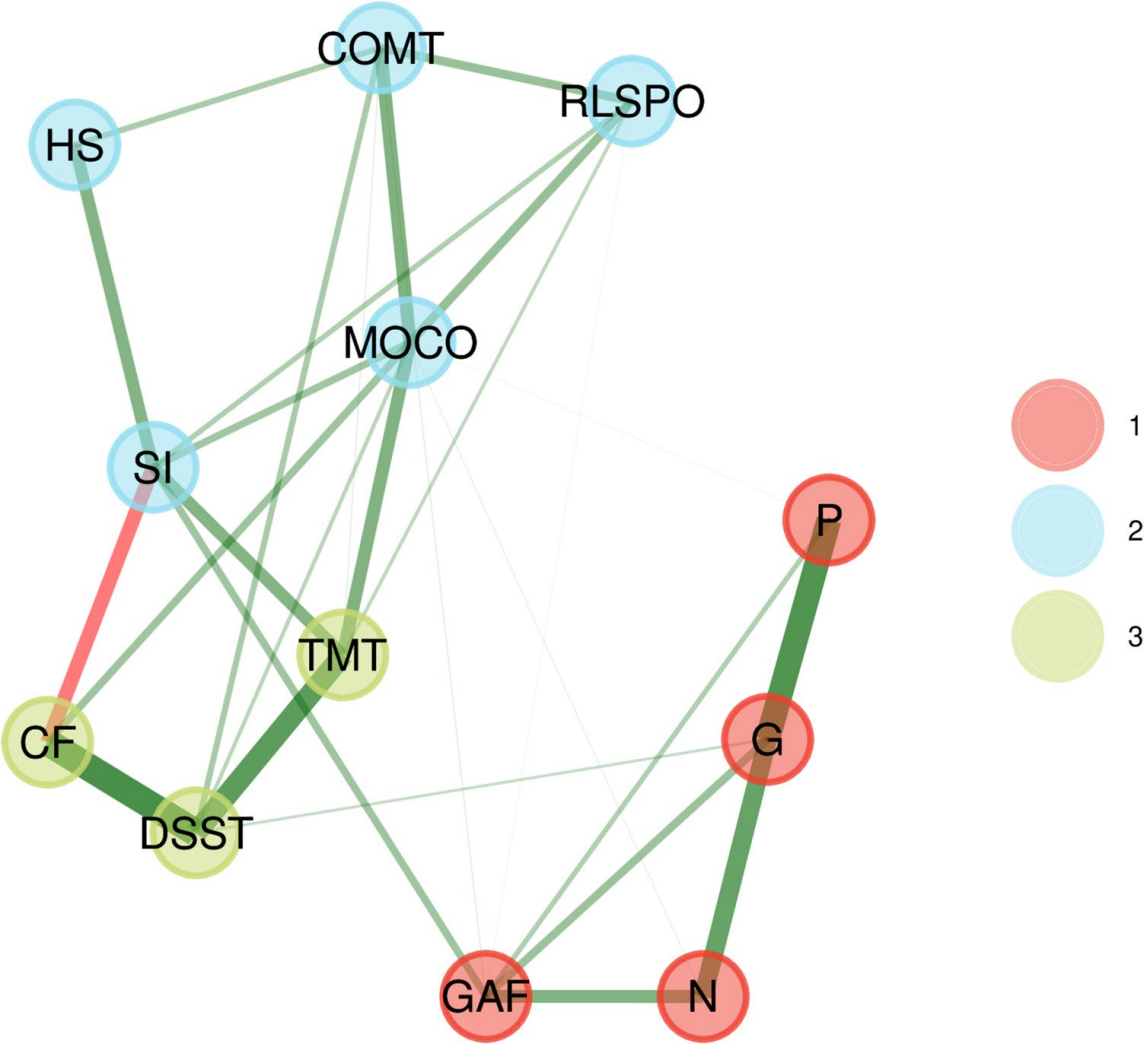
This figure highlights the bootstrapped exploratory graph analysis. This analysis revealed three communities: Cluster #1 (red) included GAF total score and the three PANSS subscores. Cluster #2 (blue) included five NSS subscores. Cluster #3 (light green) included TMT-B, DSST and CF. DSST: digit symbol substitution test; G: PANSS, general psychopathology dimension; MOCO: Neurological Soft Signs—Motor Coordination; TMT: Trail Making Test B; N: PANSS, negative psychosis dimension; COMT: Neurological Soft Signs—Complex motor tasks; GAF: Global Assessment of Functioning; SI: Neurological Soft Signs—Sensory Integration subscale; P: PANSS, positive psychosis dimension; RLSPO: Neurological Soft Signs—Right/Left and Spatial Orientation; CF: Category Fluency; HS: Neurological Soft Signs—Hard Signs

Bootstrapped EGA revealed three communities (Fig. 3): cluster #1 included GAF total score and the three PANSS subscores, cluster #2 included five NSS subscores, cluster #3 included TMT-B, DSST and CF. Item replicability was sufficient in cluster #1 and #2, yet there was low replicability in cluster #3 (Table 3). Bootstrapping dimension frequency was 0.57 for two factors and 0.39 for three factors, the median dimension was 2, and the confidence interval was 0.85–3.15. Network loadings are shown in Table 4. In total, the EGA results were deemed unstable and therefore, we refrained from repeating the EGA including age, sex, education, and medication.

**Table 3:** Item replicability corresponding to the exploratory graph analysis (EGA) community structure

	Community^a^		
	1	2	3
COMT	0.96	–	–
RLSPO	0.96	–	–
MOCO	0.90	–	0.10
HS	0.93	–	–
SI	0.87	–	0.11
PANSS-P	–	1.00	–
PANSS-G	–	1.00	–
PANSS-N	–	1.00	–
GAF	–	1.00	–
CF	0.61	–	0.40
DSST	0.61	–	0.39
TMT-B	0.64	–	0.36

*PANSS* positive and negative syndrome scale (*P* positive, *G* general, *N* negative), *GAF* global assessment of functioning, *NSS* neurological soft signs, *COMT* complex motor tasks, *MOCO* motor coordination, *SI* sensory integration, *RLSPO* right/left spatial orientation, *HS* hard signs, *CF* category fluency, *DSST* digit symbol substitution test, *TMT-B* trail making test part B

^a^Values depict the proportion of times which an item formed part of the community in the bootstrapped EGA samples

**Table 4:** Network loadings corresponding to the Exploratory Graph Analysis (EGA) Community Structure

Clinical variable	Community^a^		
	1	2	3
COMT	0.25	0.03	0.10
RLSPO	0.20	0.03	0.06
MOCO	0.34	0.03	0.25
HS	0.15	0.02	0.03
SI	0.31	0.09	0.19
PANSS-P	0.02	0.32	0.02
PANSS-G	0.02	0.55	0.03
PANSS-N	0.04	0.34	0.02
GAF	0.09	0.28	0.01
CF	0.25	0.02	0.33
DSST	0.29	0.04	0.48
TMT-B	0.31	0.01	0.28

*PANSS* positive and negative symptoms scale (*P* positive, *G* general, *N* negative)*, GAF* global assessment of functioning, *NSS* neurological soft signs, *COMT* complex motor tasks, *MOCO* motor coordination, *SI* sensory integration, *RLSPO* right/left spatial orientation, *HS* hard signs, *CF* category fluency, *DSST* digit symbol substitution test, *TMT-B* trail making test part B

^a^Values depict the influence which an item showed on forming of a specific community. Higher values indicate higher influence

### Covariate-adjusted correlation analysis

The partial correlation (C) (after log-transformation of variables that were not normally distributed) revealed that CF was significantly associated with NSS MOCO as well as NSS SI while controlling for all other variables (*r* = 0.25, *p* < 0.001 and *r* = − 0.33, *p* < 0.001, respectively; uncorrected for multiple testing). Also, TMT-B was associated with NSS SI while controlling for all other variables (*r* = 0.28, *p* < 0.001; uncorrected for multiple testing). Notably, PANSS scores were not significantly associated with NSS scores (uncorrected for multiple testing).

## Discussion

Using the network approach to psychopathology, we investigated—for the first time—the interrelationship between psychopathological symptoms, sensorimotor, cognitive, and global functioning in a transdiagnostic sample consisting of SSD, MDD, and BD patients. Two main findings emerged: First, NSS showed closer connections with TMT-B, CF and DSST than with GAF and PANSS. Second, DSST, PANSS general, and NSS MOCO scores showed the highest EI, while SI, DSST, and CF showed the highest strength. However, the EGA was unstable and could not be interpreted.

The first finding supports and extends our understanding of the relationship between sensorimotor and cognitive domains for several reasons: First, there has been prior research linking more severe hypokinetic sensorimotor abnormalities (e.g., NSS and parkinsonism) to executive functioning deficiencies in SSD [52, 53]. Second, our findings are in line with Wolf et al. [54] and confirm the precise link between the sensorimotor and cognitive domains. Our findings extend this previous work transdiagnostically. Third, the TMT-B and DSST assessments are well-known tools of cognitive performance, particularly in terms of processing speed, cognitive flexibility, and the phenomenon known as psychomotor slowing. This line of reasoning suggests that a more inclusive definition of this term may incorporate sensorimotor disorders such as NSS [55]. Accordingly, Osborne et al. [55] described that the cognitive (“psycho”) and sensorimotor sub-processes that lead to psychomotor slowing may overlap with the sub-processes of subtle sensorimotor abnormalities such as NSS. Finally, NSS and TMT-B are associated with overlapping circuitry, suggesting a biological link between sensorimotor and cognitive processes [56]. On one side, changes in the inferior frontal gyrus, paracentral gyrus, inferior parietal lobe (IPL), bilateral putamen, cerebellum, and both the superior and middle temporal gyri (STG and MTG) are associated with NSS (for a summary, see also Hirjak et al. [57, 58] and Zhao et al. [59]). On the other side, the prefrontal cortex, the IPL, and the cerebellum play also a crucial role in the execution of TMT-B [60]. This implies that a shared neurobiological process may lead to manifest sensorimotor and cognitive symptoms in SSD and MOD. Our results together with the above mentioned evidence extend our current understanding of interrelated behavior in the RDoC Matrix.

Last but not least, although there have been reports—also from our group [19]—examining the bilateral link between NSS and psychopathology as well as NSS and cognition [20, 21], the precise trilateral relationship between cognition, NSS, and psychopathology remains unclear. We interpret our results as suggesting that sensorimotor symptoms are closer to cognition than psychopathology, but this relationship does not exclude connections between NSS and psychopathology. Indeed, as shown in Table 2, PANSS positive and negative subscales showed (rather weak) partial correlations with NSS subscales. Psychopathology also showed few connections with cognitive symptoms, which is in line with assumptions that the psychopathology domain by itself (as outcome parameter) only incompletely reflects functional impairment, which is more closely connected with cognitive deficits [61].

Second, the most central nodes in terms of EI were DSST, PANSS general, and NSS MOCO scores. All nodes showed a positive value in the centrality index. In terms of strength, SI, DSST and CF showed the highest centrality. These findings are crucial for a number of reasons: First, the DSST examines processing speed, working memory, visuospatial processing, and attention. Also, another recent network analysis in SSD showed a central place for processing speed evaluated with symbol coding [42]. This said, DSST reflects the global cognitive functioning, which, when impaired, can lead to disturbances at the level of sensorimotor functions. In line with this, Morrens et al. [62] used DSST (e.g., matching time and writing time) to examine sensorimotor and cognitive slowing in SSD. Although the authors concluded that both processes are unrelated, matching time was associated with neuropsychological test results. Second, this fits in well, because MOCO also plays a central role in our network. The MOCO subscale includes five items, such as Ozeretski’s test, diadochokinesia, pronation/supination, finger-to-thumb opposition, and speech articulation. Both DSST and MOCO are based on movement execution, predominantly at the level of sensorimotor and visuospatial control. Third, disturbances of sensorimotor and spatial–visual control can lead to various psychopathological symptoms, such as somatic concerns, anxiety, depression, motor retardation, disorientation, disturbance of volition, poor impulsive control, and preoccupation, respectively [63, 64]. This could be a possible explanation for the centrality of the PANSS general scores. Fourth, as proposed by the European consensus on assessments and perspectives [26], new treatment targets for stimulation techniques need to be identified. From a network point of view, variables are interrelated and targeting the most central nodes could affect other nodes as well. Consequently the connection between sensorimotor symptoms and cognition and the high centrality of sensorimotor symptoms suggest that sensorimotor symptoms and cognitive symptoms may share aspects of pathophysiology. Specifically, the high centrality of MOCO and SI together with the previously identified neurobiological correlates of NSS may suggest NSS MOCO and SI as possible treatment targets for stimulation techniques (such as transcranial direct current stimulation or repetitive transcranial magnetic stimulation) in order to improve cognition. In addition, one may wonder whether movement exercises that train the individual items of the two subscales could also lead to an improvement in cognition. This is in accordance with previous reports showing associations between NSS and cognition in longitudinal investigations [20]. Previously reported structural correlates of NSS [25] would be the primary neurobiological target regions for these stimulation techniques. This train of thought seems particularly relevant since cognitive deficits in SSD are associated with lower global and social functioning [61, 65] and are difficult to treat so far. Yet, while structural correlates of NSS have been repeatedly demonstrated in SSD [25, 66–69], we are not aware of such data in BD or MDD. When interpreting our results, it is important to bear in mind that there may not be a direct link between our non-direct, non-causal network analysis and neurobiological pathophysiology.

Taken together, complex phenomena (such as cognitive, psychopathological, and sensorimotor symptoms) may be best described at the systems level [13, 70–72]. More precisely, a shift from focusing on individual components to studying the organization of the system’s components seems promising [13, 70–72]. Network analysis provides tools to identify a system’s components and their relationships [70]. In this context, the particular benefit of network analysis techniques may be provided by their focus on patterns of pairwise conditional relationships as well as by enabling powerful visualizations of those patterns [70]. Furthermore, previous studies have established relationships between the sensorimotor and cognitive domain [52, 53]. However, the methods applied in these studies were limited: they were not able to simultaneously take into account the dynamic effects of other relevant variables on the relationship of interest as network analysis can. Also, associations between the sensorimotor and psychopathological domains have been reported as well [19]. Yet, how these domains interact simultaneously with each other has not been investigated so far. Here, our network analysis for the first time models the interactions between the sensorimotor and cognitive domains while accounting for psychopathological symptoms and global functioning. Our results suggest that there is a closer connection between the sensorimotor and cognitive domains than between the sensorimotor and psychopathological domains. Furthermore, the reported association between the sensorimotor and cognitive domains after accounting for psychopathological symptoms and global functioning may implicate several future investigative steps: First, the close connection between the sensorimotor and cognitive domains on the level of clinical tests suggest overlapping pathophysiology between both constructs [25, 60, 73]. Longitudinal MRI studies with several network analyses sequentially excluding psychopathological symptoms and global functioning may shed more light on the precise relationship between sensorimotor and cognitive symptoms. Another road may be—in accordance with previous network analysis literature [70, 71, 74]—to consider either sensorimotor symptoms or cognitive symptoms as treatment targets and investigate the effects on both domains after pharmacological and non-pharmacological (stimulation techniques) interventions.

### Strengths and limitations

The study sample size, a solid theoretical framework, and the use of two network indices, such as EI and strength centrality, are the main strengths of this study. However, this study also has limitations: First, sample size of BD and MDD subgroups was limited, which could imply that our results may have been more influenced by our SSD subgroup. Due to small group sizes in our MOD participants, we were not able to perform the network analyses in this subgroup, thus uncertainty remains whether our findings truly extend to MOD. Yet, in separate analyses including only SSD participants (*n* = 174), the results remained comparable. Still, the imbalance in the examined groups concerning the diagnosis could lead to sampling bias, making it difficult to generalize the findings to the broader population. Therefore, the question as to whether sensorimotor symptoms are really a transdiagnostic therapeutic target needs to be examined in future studies including larger and more balanced diagnostic groups. Second, by employing cross-sectional data, we cannot make inferences about long-term relationships between psychopathology, sensorimotor, and cognitive symptoms and we advocate longitudinal studies which could better test for causality. Third, we did not employ second-generation negative symptom scales such as the BNSS. However, our focus in this project was to analyze the entire psychopathological domain, for which PANSS remains the most widely used scale in SSD and other psychiatric disorders such as MOD (please refer to PANSS general symptoms). Fourth, we are aware of the previous network analyses [14, 15, 75, 76] which examined data on several levels ranging from total scores to sub-scores to single items level. The authors stated that more knowledge could be accumulated through the multilevel analysis. The study by Fried et al. [77] corroborate this view and performed network analyses on several levels. The authors [77] reported that relationships between depression and inflammation were strongly attenuated after controlling for BMI. They also concluded that decomposing sum scores may lead to reduced reliability [77]. Therefore, we assume that our network analysis could reveal more detailed insights if performed on several levels. However, in our study, we were interested in the interplay between psychopathology, sensorimotor, cognitive and global functioning more globally. Thus, we opted for subscores/subscales rather than single-item level scores. In addition, investigating many individual features (i.e., including more nodes in the network) makes network estimation more complex, requiring larger study groups in order to yield stable networks and consequently, we refrained from these analyses. Last but not least, our hypotheses were focused on investigations of the relationships between the different domains and not individual items.

## Conclusion

This is the first study that sought to investigate the pattern of connections among a wide array of functional domains, such psychopathological symptoms, sensorimotor, cognitive, and global functioning in a transdiagnostic sample consisting SSD and MOD patients. Aspects of the pathophysiology underlying both SSD and MOD are likely shared, given the close relationship between NSS and cognitive functioning and the high centrality of sensorimotor symptoms. However, given that a significant proportion of the study participants had SSD, further research with more balanced diagnostic groups is required to determine whether sensorimotor symptoms truly represent a transdiagnostic therapeutic target.

## Supplementary Information

Below is the link to the electronic supplementary material.

Supplementary file1 (DOCX 2776 KB)

## Data Availability

The data presented in this study are available from the corresponding author on reasonable request.
