# Correction: Deciphering the interplay between psychopathological symptoms, sensorimotor, cognitive and global functioning: a transdiagnostic network analysis

**DOI:** 10.1007/s00406-024-01824-w

**Published:** 2024-05-13

**Authors:** Stefan Fritze, Geva A. Brandt, Sebastian Volkmer, Jonas Daub, Maria Krayem, Jacqueline Kukovic, Emanuel Schwarz, Urs Braun, Georg Northoff, Robert Christian Wolf, Katharina M. Kubera, Andreas Meyer-Lindenberg, Dusan Hirjak

**Affiliations:** 1 Department of Psychiatry and Psychotherapy, Central Institute of Mental Health, Medical Faculty Mannheim, University of Heidelberg, 68159 Mannheim, Germany; 2 Hector Institute for Artificial Intelligence in Psychiatry, Central Institute of Mental Health, Medical Faculty Mannheim, Heidelberg University, Mannheim, Germany; 3 Mind, Brain Imaging and Neuroethics Research Unit, The Royal’s Institute of Mental Health Research, University of Ottawa, Ottawa, ON Canada; 4 Center for Psychosocial Medicine, Department of General Psychiatry, University of Heidelberg, Heidelberg, Germany; 5 German Centre for Mental Health (DZPG), Partner Site Heidelberg/Mannheim/Ulm, Mannheim, Germany

**Correction: European Archives of Psychiatry and Clinical Neuroscience** 10.1007/s00406-024-01782-3

In this article, the affiliation 5 (German Centre for Mental Health (DZPG), Partner Site Heidelberg/Mannheim/Ulm, Mannheim, Germany) was not assigned to the authors Emanuel Schwarz, Urs Braun, Andreas Meyer-Lindenberg and Dusan Hirjak.

Also, the affiliation 5 was incorrectly published as Present Address: German Centre for Mental Health (DZPG), Partner Site Heidelberg/Mannheim/Ulm, Mannheim, Germany but should have been German Centre for Mental Health (DZPG), Partner Site Heidelberg/Mannheim/Ulm, Mannheim, Germany.

The original article has been corrected.

