# Supplementary material for: Deciphering the interplay between psychopathological symptoms, sensorimotor, cognitive and global functioning: a transdiagnostic network analysis
Source: Eur Arch Psychiatry Clin Neurosci. 2024 Mar 20;274(7):1625–37. doi: 10.1007/s00406-024-01782-3 (PMC11422259; doi:10.1007/s00406-024-01782-3)
Supplement: Supplementary file 1 — Supplementary file1 (DOCX 2776 KB) [file 406_2024_1782_MOESM1_ESM.docx]

**Supplementary material:**

**Supplementary table 1.** Clinical and demographic variables of SSD, MDD and BD study participants.

| Variable | SSD (n=174) | MDD(n=26) | BD(n=12) | *KW/X^2^* | *df* | *p* | Dunn test |
| --- | --- | --- | --- | --- | --- | --- | --- |
| Age | 37.55±11.74 | 37.11±14.19 | 44.42±13.86 | 3.419 | 2 | 0.181 | - |
| Sex (m/f) | 84/90 | 16/10 | 4/8 | 2.850 | 2 | 0.241 | - |
| Education (years) | 13.18±2.91 | 13.85±2.71 | 13.83±2.55 | 2.149 | 2 | 0.341 | - |
| PANSS Positive | 15.5±6.5 | 8.8±1.8 | 9.2±1.9 | 43.16 | 2 | **<0.001** | **SSD>MDD (p<0.001); SSD>BD (p<0.001)** |
| PANSS Negative | 17.2±7.4 | 15.2±6.6 | 15.1±7.9 | 2.733 | 2 | 0.255 | - |
| PANSS General | 34.5±10.3 | 31.0±6.6 | 31.4±8.9 | 3.810 | 2 | 0.149 | - |
| NSS MOCO | 7.6±4.1 | 4.0±2.0 | 5.9±3.2 | 23.116 | 2 | **<0.001** | **SSD>MDD (p<0.001)** |
| NSS SI | 3.2±1.9 | 3.9±1.4 | 3.3±1.1 | 4.668 | 2 | 0.097 | - |
| NSS COMT | 3.5±2.3 | 2.3±2.1 | 3.6±2.0 | 7.880 | 2 | **0.019** | **SSD>MDD (p=0.006)** |
| NSS RLSPO | 2.9±2.5 | 2.3±1.7 | 2.6±1.7 | 0.965 | 2 | 0.617 | - |
| NSS HS | 3.2±1.9 | 3.2±1.7 | 3.1±2.3 | 0.024 | 2 | 0.988 | - |
| GAF* | 53.44±16.9 | 55.2±13.0 | 64.7±21.2 | 5.147 | 2 | 0.076 | - |
| CF* | 39.3±17.4 | 11.8±4.4 | 30.4±17.5 | 52.311 | 2 | **<0.001** | **SSD>MDD (p<0.001); MDD<BD (p=0.004)** |
| DSST* | 50.8±20.9 | 23.3±14.6 | 47.9±23.2 | 34.312 | 2 | **<0.001** | **SSD>MDD (p<0.001); MDD<BD (p=0.003)** |
| TMT-B (seconds) | 112.8±64.4 | 74.15±31.2 | 98.2±44.6 | 12.504 | 2 | **0.002** | **SSD>MDD (p<0.001)** |

Data are mean ± standard deviation and Kruskal-Wallis or Chi-square test as well as post-hoc Dunn test.

Abbreviations: *SSD* Schizophrenia Spectrum Disorders, *MDD* Major Depressive Disorder, *BD* Bipolar Disorder, *SD* Standard Deviation, *PANSS* Positive and Negative Symptoms Scale, *NSS* Neurological Soft Signs, *MOCO* Motor Coordination, *SI* Sensory Integration, *RLSPO* Right/Left Spatial Orientation, *HS* Hard Signs, *CF* Category Fluency, *DSST* Digit Symbol Substitution Test, *TMT-B* Trail Making Test part B.

*GAF, CF and DSST are reverse coded values.

*Network stability*

We employed bootstrapping as implemented in the *bootnet* package with 95% confidence intervals and examined edge stability (supplementary figure 1) as well as expected influence stability as a measure of centrality (supplementary figure 2). Here, we calculated correlation stability coefficient defined by the proportion of participants which can be excluded from the original sample while keeping a correlation of at least 0.7 for centrality. The recommended correlation stability coefficient of 0.25 was clearly exceeded in our analyses of the whole sample (n=212) as well as the SSD sample (n=174) which were consequently considered stable.

**Supplementary figure 1.** Edge stability as assessed by bootstrapping.


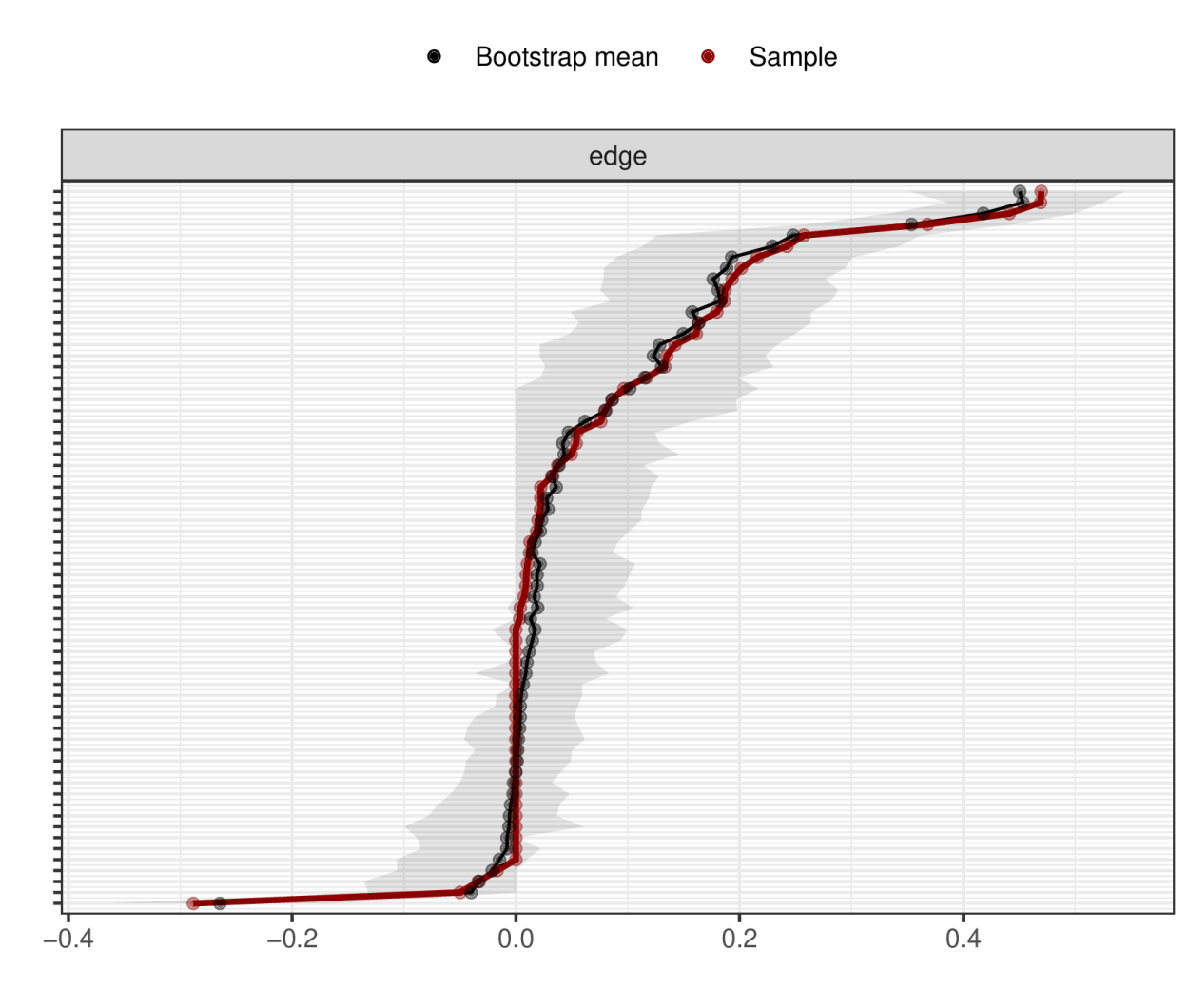


Edge-weights are sorted in increasing order (red). Grey areas represent 95% confidence intervals. Edge-edge relationships are depicted on the y-axis with labels omitted.

**Supplementary figure 2.** Case-dropping procedure to evaluate stability of expected influence and strength.


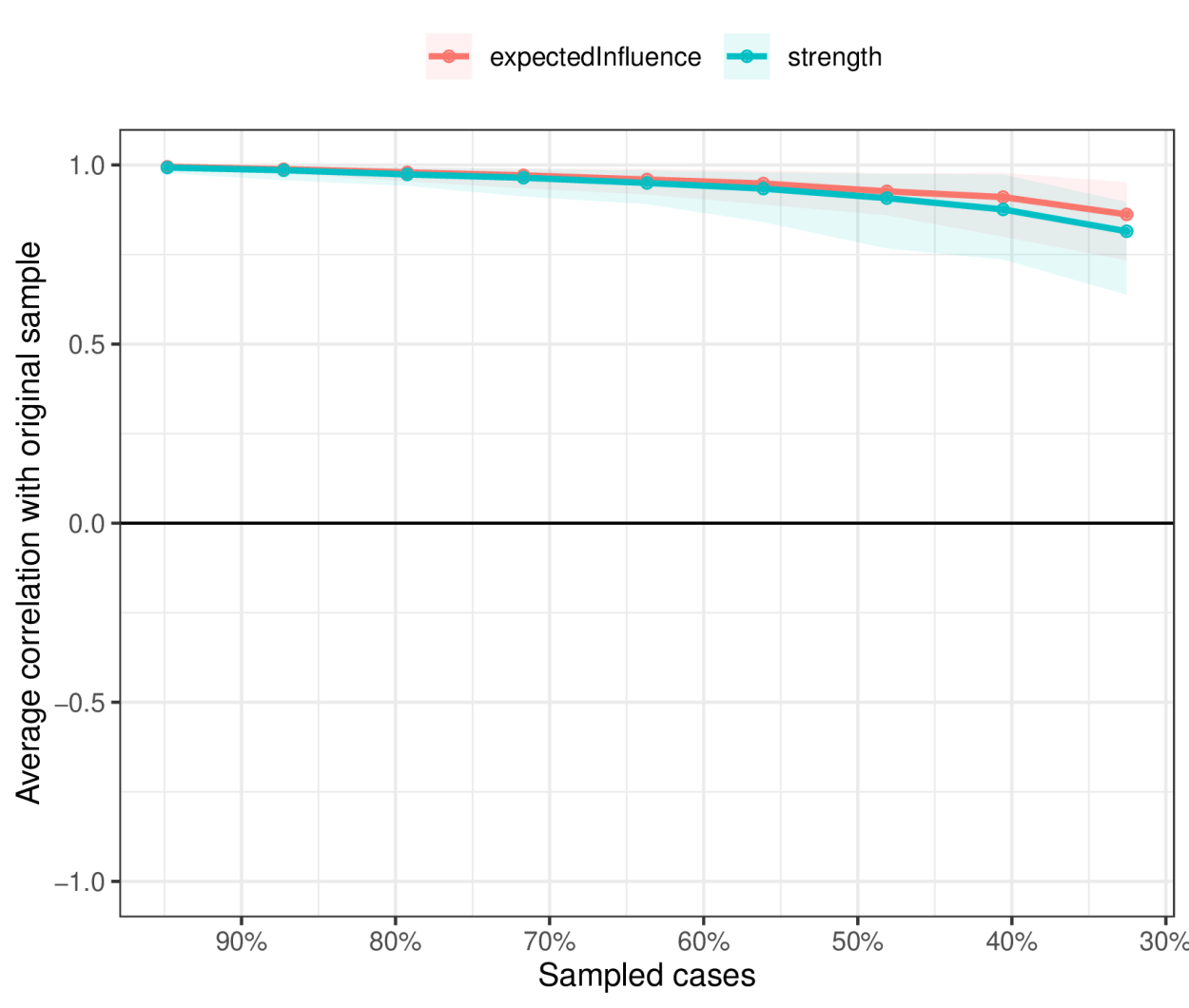


Correlations between expected influence and strength values (whole sample) and newly estimated expected influence in subgroups with decreasing participant percentages. Percentages of sampled participants are depicted on the x-axis. Average correlations are reported on the y-axis.

**Supplementary figure 3.** Centrality difference test (expected influence).


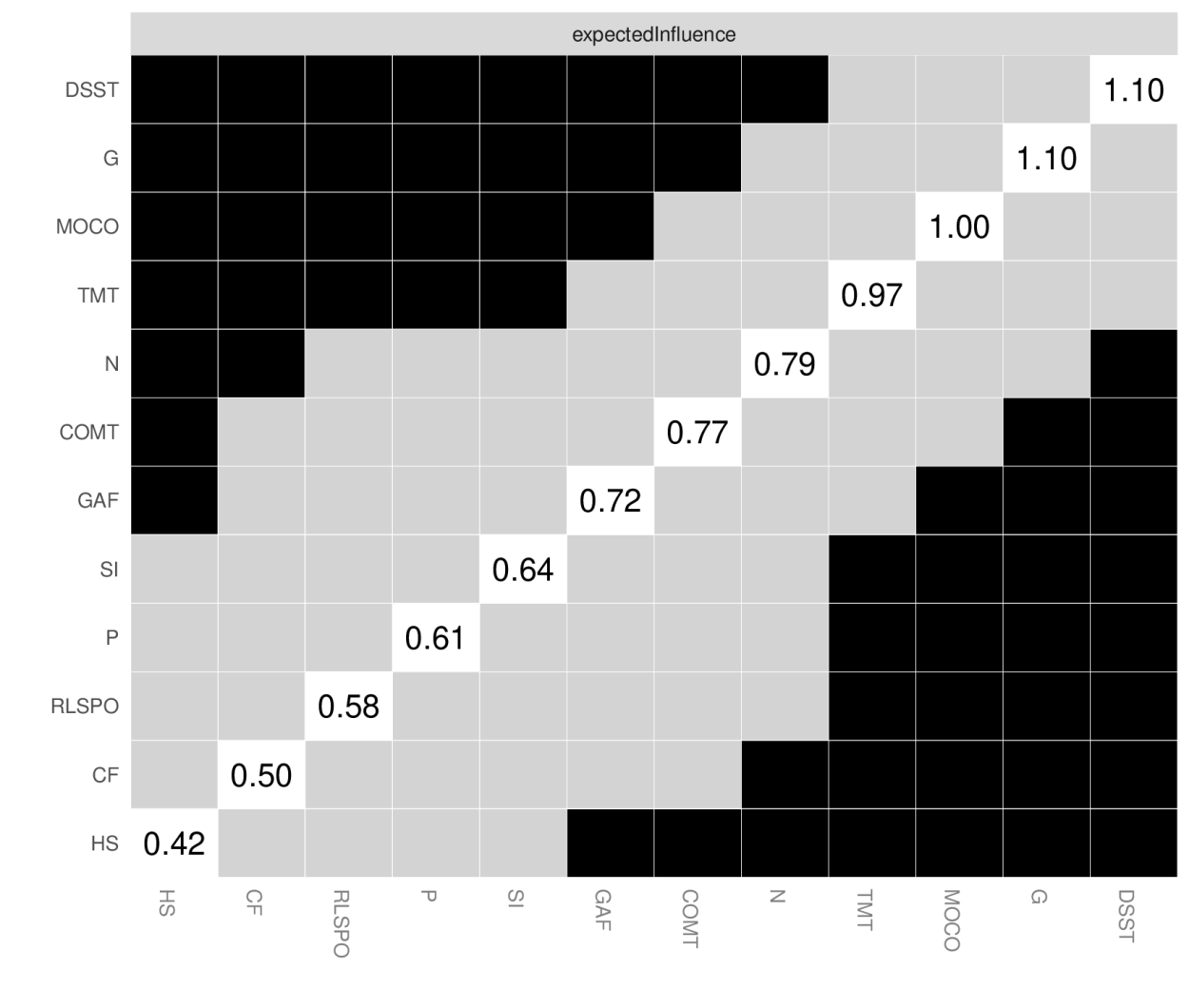


Black tiles represent significantly different centralities. Grey tiles represent non-significantly different centralities. MOCO centrality was not significantly different from centrality of either DSST, PANSS general or TMT-B.

**Supplementary figure 4.** Centrality difference test (strength)


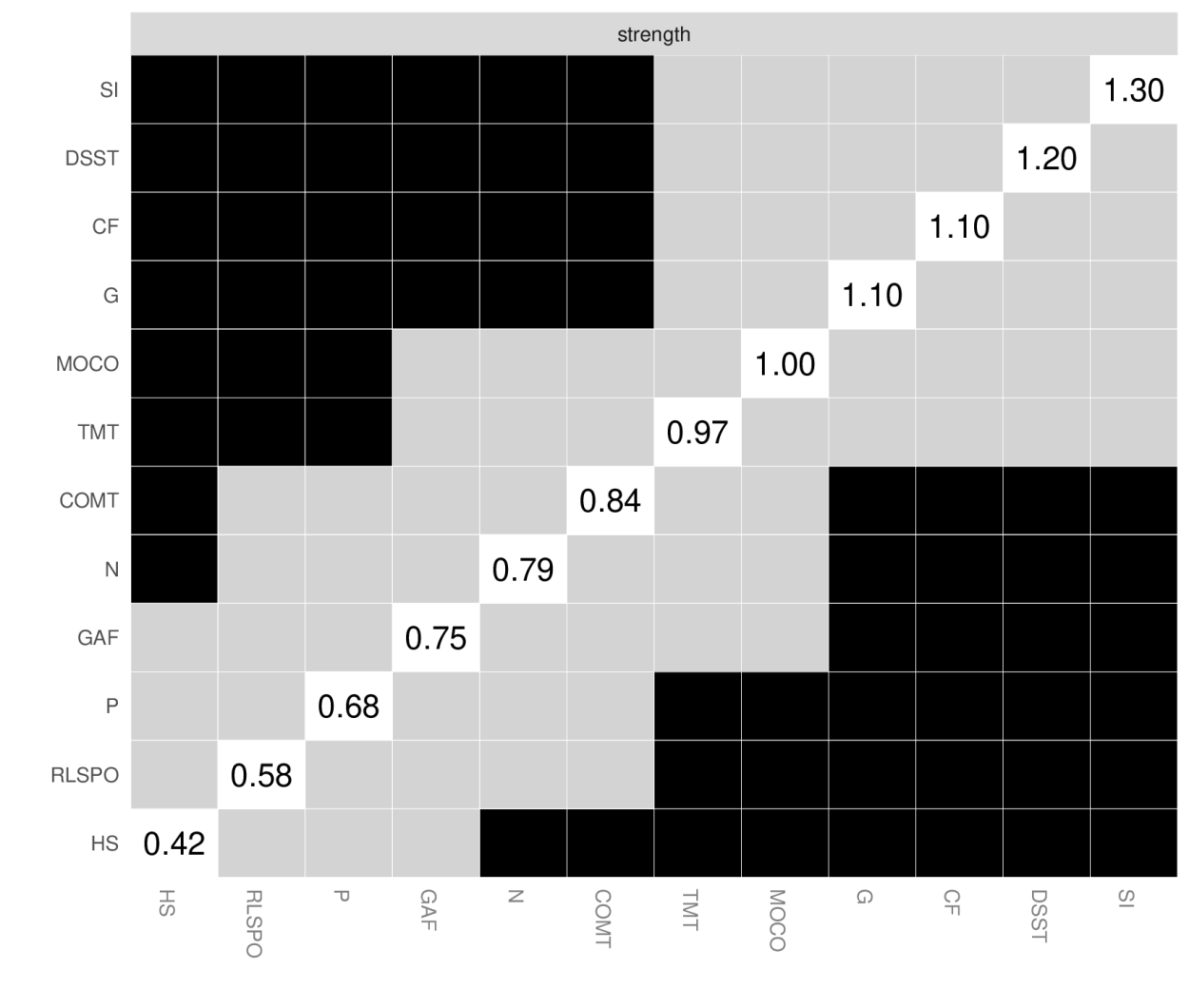


Black tiles represent significantly different centralities. Grey tiles represent non-significantly different centralities. SI centrality was not significantly different from centrality of either DSST, CF, PANSS general, MOCO or TMT-B.

**Supplementary figure 5.** Edge-weight difference test.


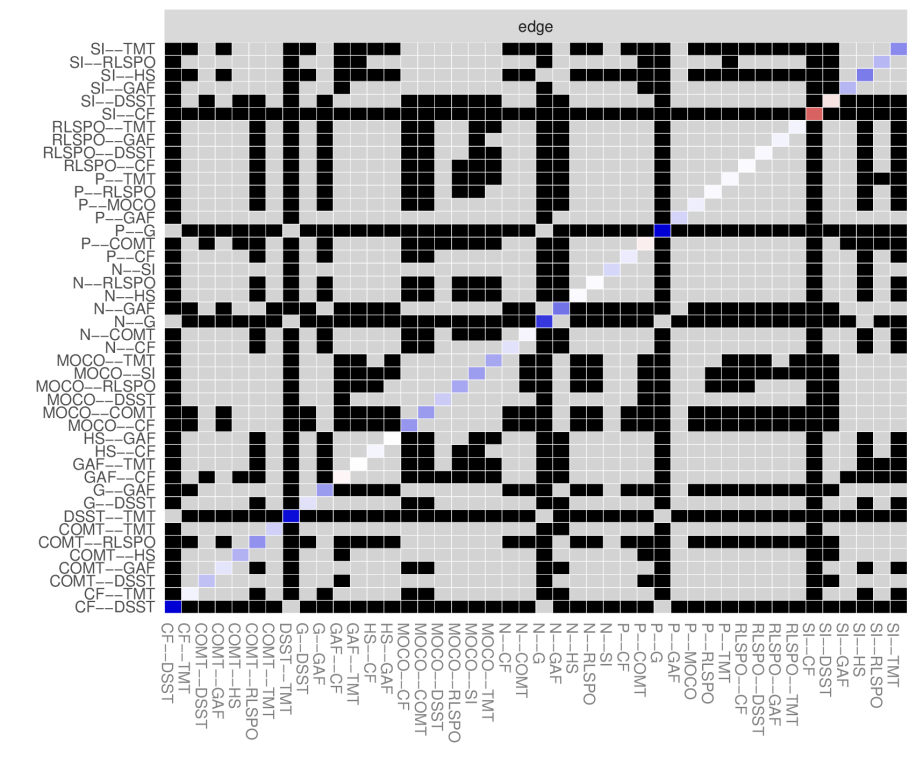


Black tiles represent significantly different edge-weights. Grey tiles represent non-significantly different edge-weights.

In order to investigate the influence of distinct diagnostic groups on the results, we performed the network analyses again by including only SSD participants (n=174). Re-analyses with MDD (n=26) or BD (n=12) subgroups were not deemed feasible due to their smaller size which may lead to unstable networks.

**Supplementary figure 6.** Network plot including only SSD participants (n=174).


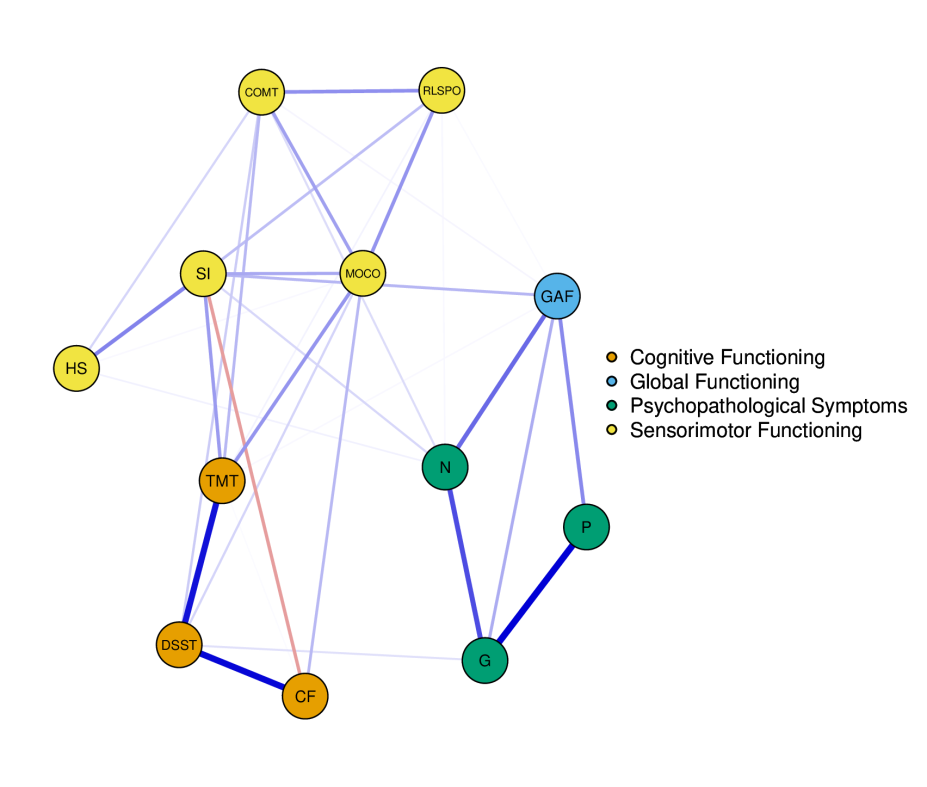


**Supplementary figure 7.** Centrality measured by Expected Influence as well as Strength in SSD participants (n=174).


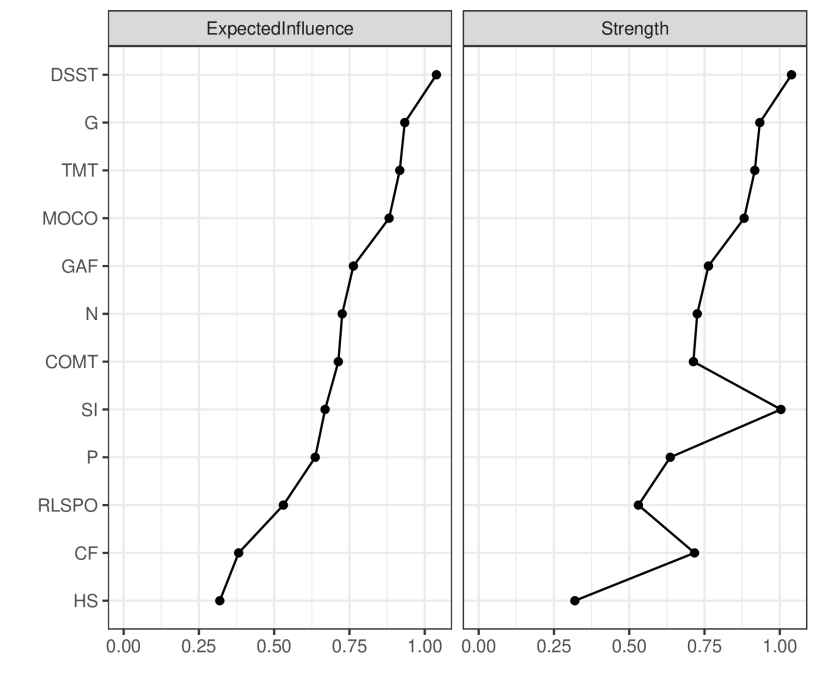


**Supplementary figure 8.** Edge stability as assessed by bootstrapping in SSD participants (n=174).


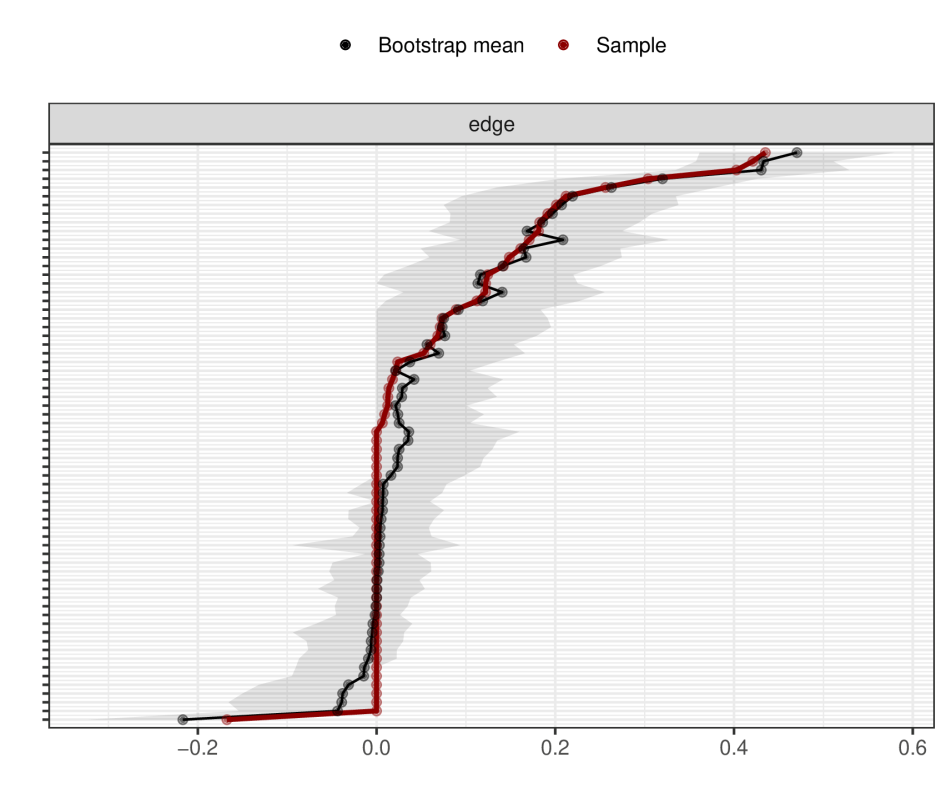


Edge-weights are sorted in increasing order (red). Grey areas represent 95% confidence intervals. Edge-edge relationships are depicted on the y-axis with labels omitted.

**Supplementary figure 9.** Case-dropping procedure to evaluate stability of expected influence and strength.


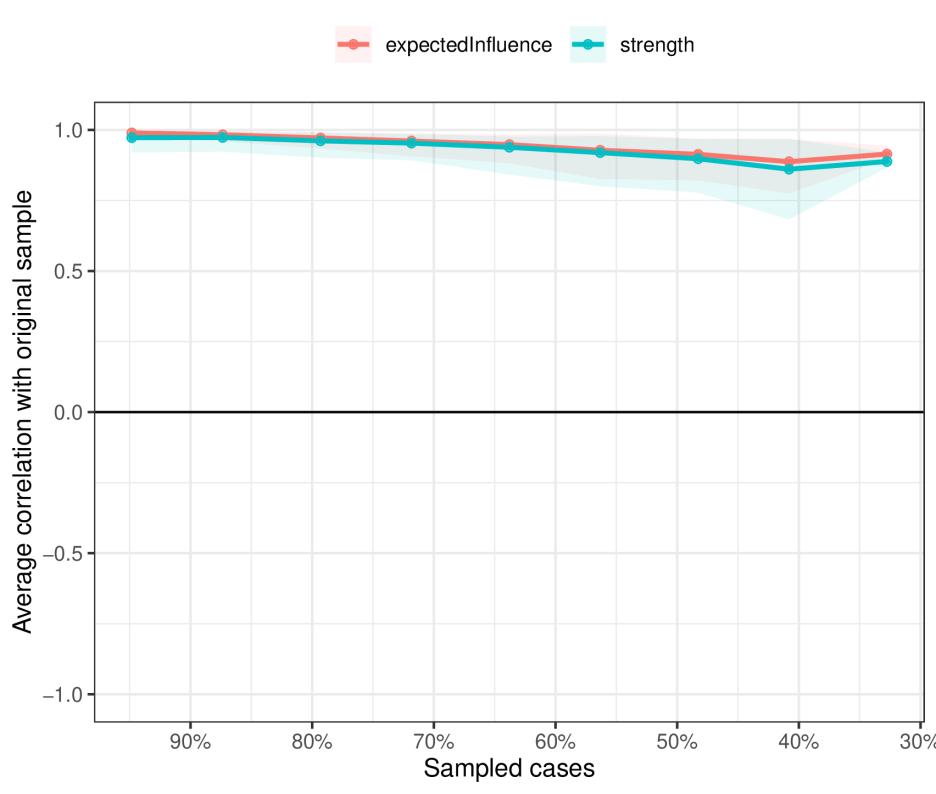


Correlations between expected influence and strength values in SSD participants (n=174) and newly estimated expected influence in subgroups with decreasing participant percentages. Percentages of sampled participants are depicted on the x-axis. Average correlations are reported on the y-axis.

**Supplementary figure 10.** Centrality difference test.


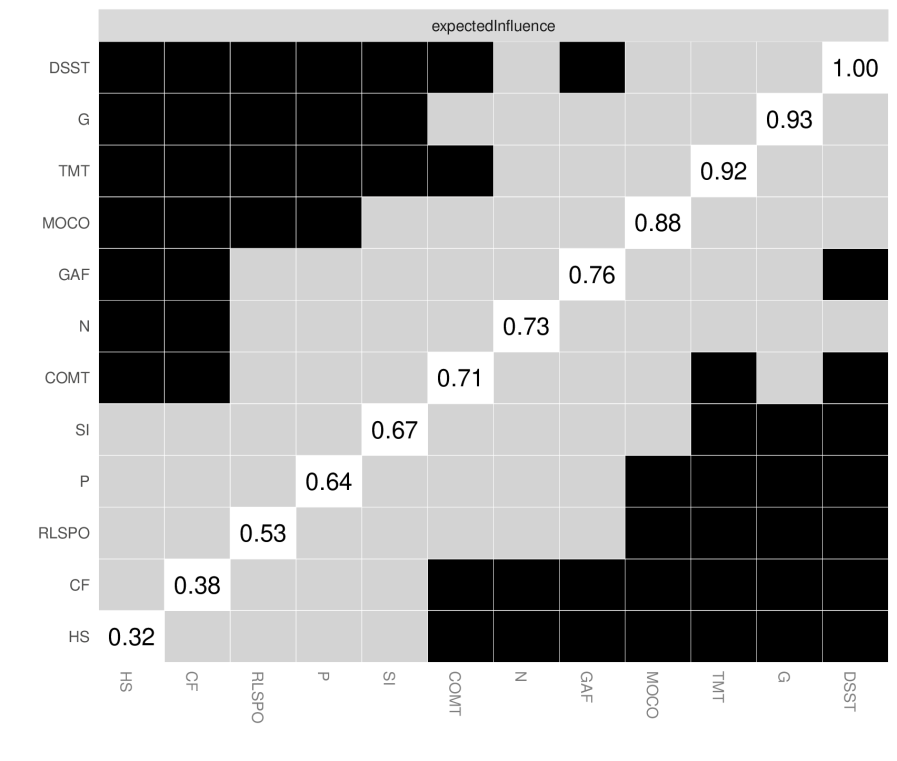


Black tiles represent significantly different centralities. Grey tiles represent non-significantly different centralities.

**Supplementary figure 11.** Centrality difference test.


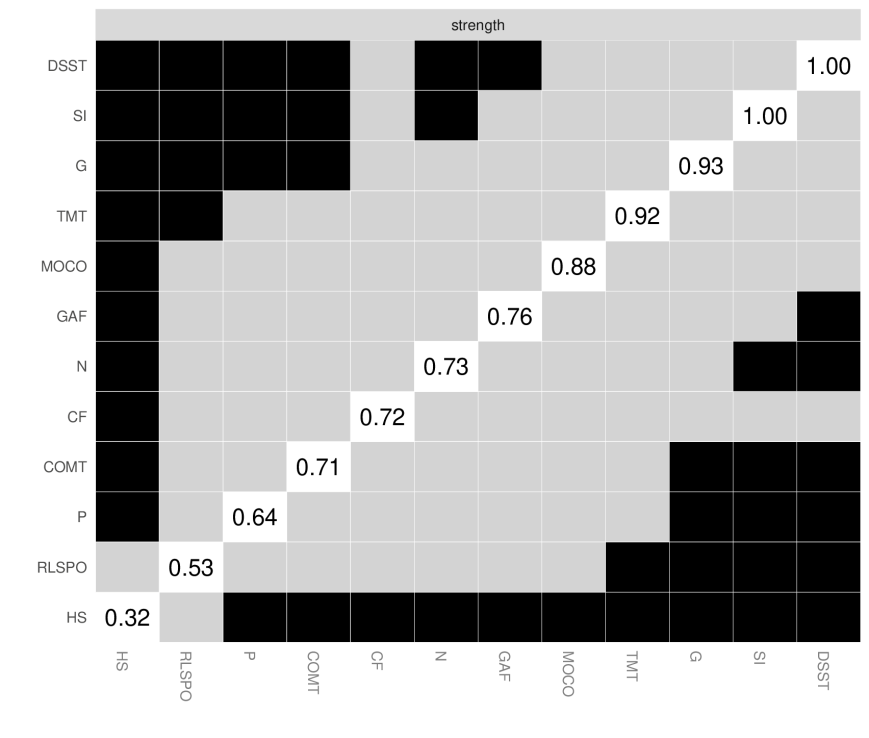


Black tiles represent significantly different centralities. Grey tiles represent non-significantly different centralities.

**Supplementary figure 12.** Edge-weight difference test.


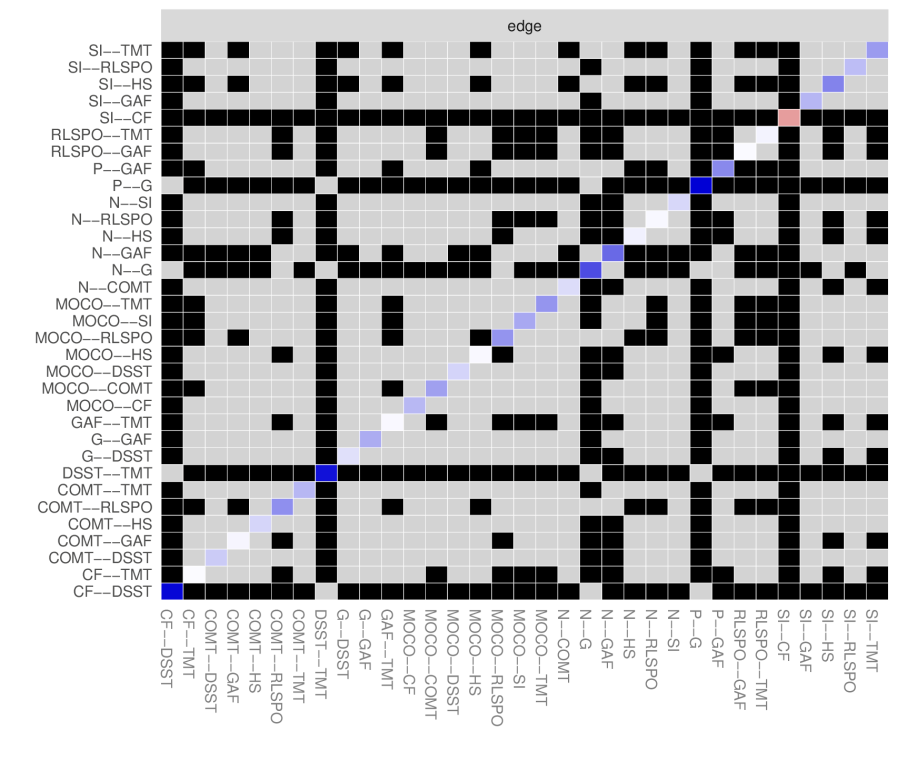


Black tiles represent significantly different edge-weights. Grey tiles represent non-significantly different edge-weights.

**Supplementary figure 13.** Network plot (n=212) including four covariates age, sex, education, and medication.


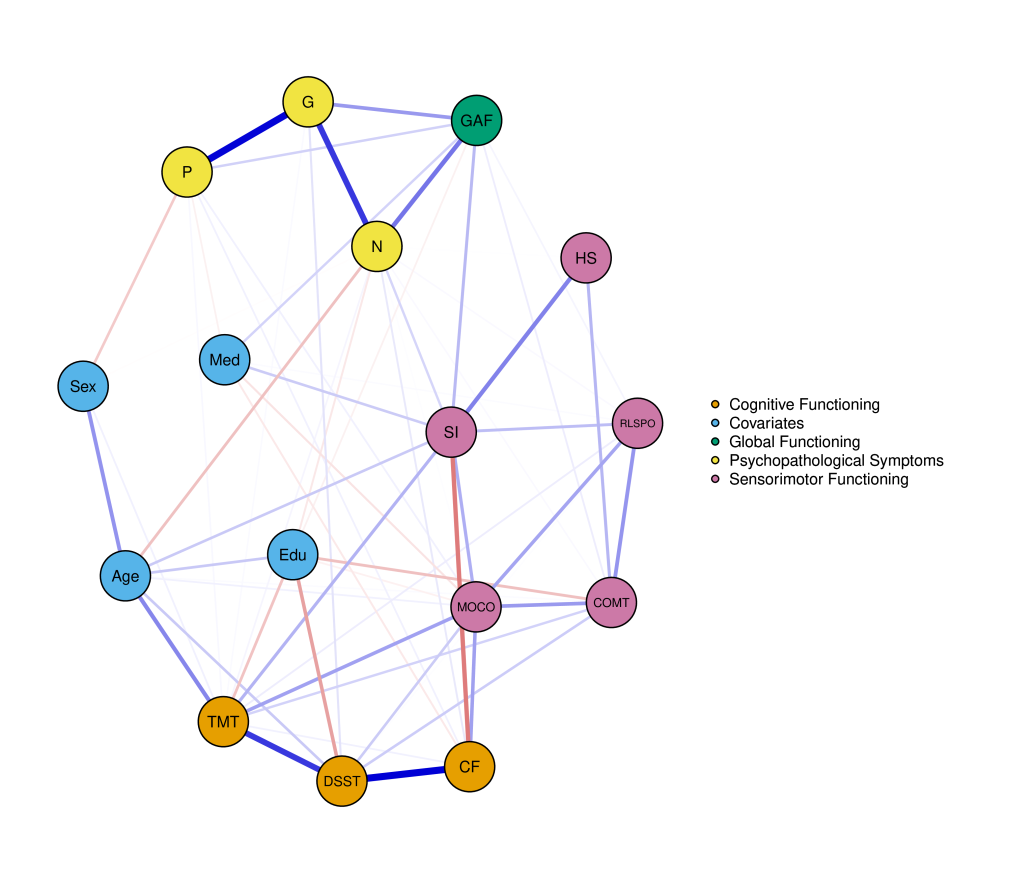


**Supplementary figure 14.** Centrality measured by Expected Influence as well as Strength in all participants (n=212), including four covariates age, sex, education, medication.


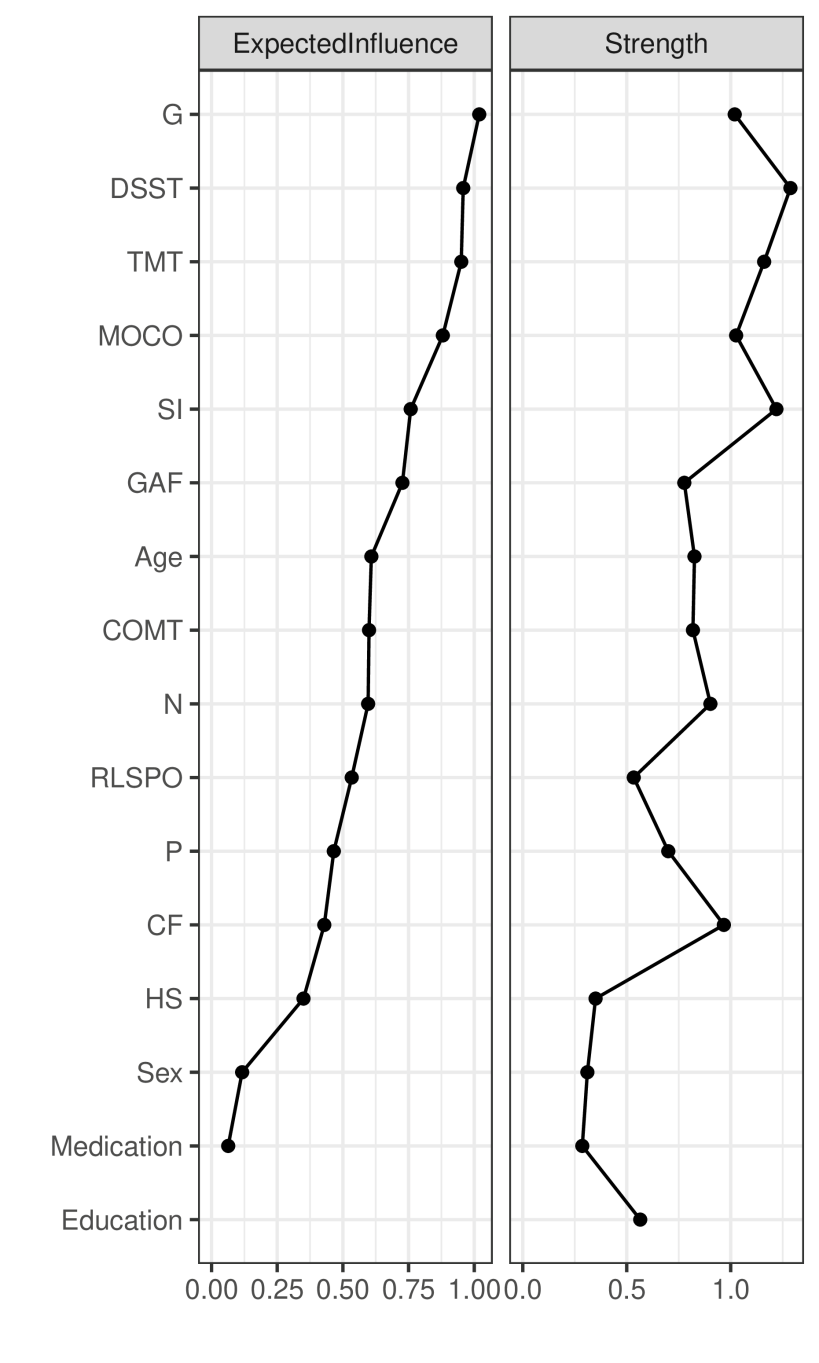


**Supplementary figure 15.** Case-dropping procedure to evaluate stability of expected influence and strength in all participants (n=212) including four covariates age, sex, education, medication.


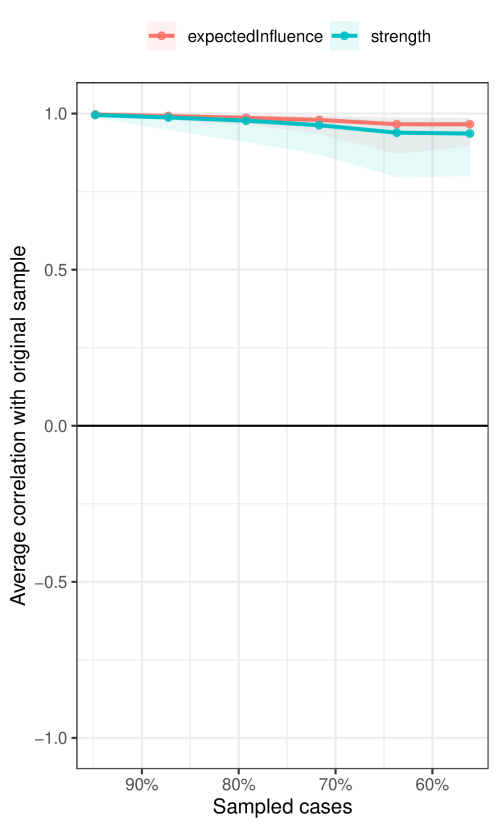


Correlations between expected influence and strength values in all participants (n=212) and newly estimated expected influence in subgroups with decreasing participant percentages. Percentages of sampled participants are depicted on the x-axis. Average correlations are reported on the y-axis.

**Supplementary figure 16.** Edge stability as assessed by bootstrapping in all participants (n=212) including four covariates age, sex, education, medication.


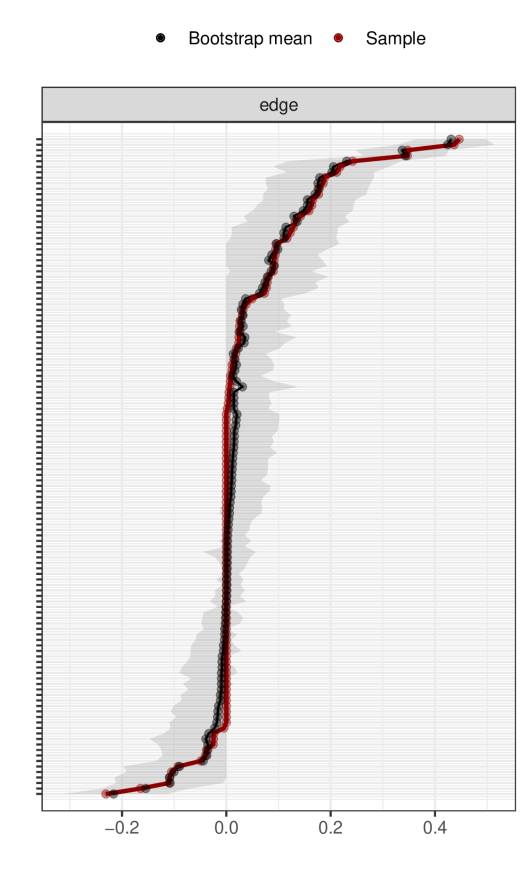


Edge-weights are sorted in increasing order (red). Grey areas represent 95% confidence intervals. Edge-edge relationships are depicted on the y-axis with labels omitted.

**Supplementary table 2.**

|  | **P** | **N** | **G** | **MOCO** | **SI** | **COMT** | **RLSPO** | **HS** | **GAF** | **CF** | **DSST** | **TMT** | **Age** | **Sex** | **Edu** | **Med** |
| --- | --- | --- | --- | --- | --- | --- | --- | --- | --- | --- | --- | --- | --- | --- | --- | --- |
| **P** | 0 | 0 | 0.44 | 0.03 | 0 | 0 | 0 | 0 | 0.08 | 0.02 | 0 | 0.01 | 0 | -0.09 | 0 | -0.02 |
| **N** | 0 | 0 | 0.35 | 0.01 | 0.07 | 0.01 | 0.01 | 0.004 | 0.24 | 0.04 | 0 | 0.02 | -0.11 | -0.005 | -0.04 | 0 |
| **G** | 0.44 | 0.35 | 0 | 0 | 0 | 0 | 0 | 0 | 0.18 | 0 | 0.05 | 0.008 | 0 | 0 | 0 | 0 |
| **MOCO** | **0.03** | **0.005** | **0** | 0 | 0.14 | 0.18 | 0.16 | 0 | 0 | **0.17** | **0.09** | **0.16** | **0.02** | **0** | **-0.02** | **-0.05** |
| **SI** | **0** | **0.07** | **0** | 0.14 | 0 | 0 | 0.12 | 0.22 | 0.12 | **-0.23** | **0** | **0.13** | **0.09** | **0** | **0** | **0.09** |
| **COMT** | 0 | 0.01 | 0 | 0.18 | 0 | 0 | 0.19 | 0.13 | 0.03 | 0 | 0.09 | 0.08 | 0.01 | 0 | -0.11 | 0 |
| **RLSPO** | 0 | 0.01 | 0 | 0.16 | 0.12 | 0.19 | 0 | 0 | 0.02 | 0 | 0.005 | 0.03 | 0 | 0 | 0 | 0.007 |
| **HS** | 0 | 0.004 | 0 | 0 | 0.22 | 0.13 | 0 | 0 | 0 | 0 | 0 | 0 | 0 | 0 | 0 | 0 |
| **GAF** | 0.08 | 0.24 | 0.18 | 0 | 0.12 | 0.03 | 0.02 | 0 | 0 | 0 | 0 | 0.002 | 0 | 0 | -0.03 | 0.08 |
| **CF** | 0.02 | 0.04 | 0 | 0.17 | -0.23 | 0 | 0 | 0 | 0 | 0 | 0.45 | 0.02 | 0 | 0 | 0 | -0.04 |
| **DSST** | 0 | 0 | 0.05 | 0.09 | 0 | 0.09 | 0.005 | 0 | 0 | 0.45 | 0 | 0.35 | 0.09 | 0 | -0.16 | 0 |
| **TMT** | 0.01 | 0.02 | 0.008 | 0.16 | 0.13 | 0.08 | 0.03 | 0 | 0.002 | 0.02 | 0.35 | 0 | 0.21 | 0.03 | -0.10 | 0 |
| **Age** | 0 | -0.11 | 0 | 0.02 | 0.09 | 0.008 | 0 | 0 | 0 | 0 | 0.09 | 0.21 | 0 | 0.19 | 0.10 | 0 |
| **Sex** | -0.09 | -0.005 | 0 | 0 | 0 | 0 | 0 | 0 | 0 | 0 | 0 | 0.03 | 0.19 | 0 | 0 | 0 |
| **Edu** | 0 | -0.04 | 0 | -0.02 | 0 | -0.11 | 0 | 0 | -0.03 | 0 | -0.16 | -0.10 | 0.10 | 0 | 0 | 0 |
| **Med** | -0.02 | 0 | 0 | -0.05 | 0.09 | 0 | 0.007 | 0 | 0.08 | -0.04 | 0 | 0 | 0 | 0 | 0 | 0 |

*Network* partial correlations between variables in our study sample (n=212) including four covariates: age, sex, education, medication. Higher values indicate stronger associations between variables. Connections between NSS (particularly MOCO and SI) are stronger with cognition than with psychopathology.

Abbreviations: *PANSS* Positive and Negative Symptoms Scale, *NSS* Neurological Soft Signs, *P* *PANSS* Positive, *N* *PANSS* Negative, *G* *PANSS* General, *MOCO* Motor Coordination, *SI* Sensory Integration, *RLSPO* Right/Left Spatial Orientation, *HS* Hard Signs, *GAF* Global Assessment of Functioning Scale, *CF* Category Fluency, *DSST* Digit Symbol Substitution Test, *TMT-B* Trail Making Test part B.

**Supplementary table 3.**

| ***r*** |  |  |  |  |  |  |  |  |  |  |  |  |  |  |  |
| --- | --- | --- | --- | --- | --- | --- | --- | --- | --- | --- | --- | --- | --- | --- | --- |
|  | **P** | **N** | **G** | **MOCO** | **SI** | **COMT** | **RLSPO** | **HS** | **GAF** | **CF** | **DSST** | **TMT** | **Age** | **Edu** | **Med** |
| **P** | 1 | -0.06 | 0.52 | 0.097 | 0.003 | -0.11 | 0.09 | 0.03 | 0.08 | 0.08 | -0.06 | 0.05 | -0.04 | 0.04 | -0.14 |
| **N** | -0.06 | 1 | 0.45 | 0.03 | 0.14 | 0.03 | 0.04 | 0.02 | 0.19 | 0.07 | -0.01 | 0.02 | -0.23 | -0.07 | 0.03 |
| **G** | 0.52 | 0.45 | 1 | -0.01 | -0.05 | 0.01 | -0.13 | -0.05 | 0.22 | -0.08 | 0.14 | -0.04 | 0.12 | 0.01 | 0.10 |
| **MOCO** | 0.097 | 0.03 | -0.01 | 1 | 0.28 | 0.25 | 0.21 | 0.004 | -0.05 | 0.25 | 0.05 | 0.10 | 0.05 | -0.05 | -0.10 |
| **SI** | 0.003 | 0.14 | -0.05 | 0.28 | 1 | -0.05 | 0.12 | 0.32 | 0.13 | -0.33 | -0.12 | 0.28 | 0.14 | 0.04 | 0.07 |
| **COMT** | -0.11 | 0.03 | 0.01 | 0.25 | -0.05 | 1 | 0.21 | 0.21 | 0.07 | -0.05 | 0.08 | 0.04 | 0.07 | -0.14 | -0.07 |
| **RLSPO** | 0.09 | 0.04 | -0.13 | 0.21 | 0.12 | 0.21 | 1 | -0.10 | 0.06 | -0.06 | 0.11 | -0.002 | -0.05 | 0.06 | 0.15 |
| **HS** | 0.03 | 0.02 | -0.05 | 0.004 | 0.32 | 0.21 | -0.10 | 1 | 0.03 | 0.08 | 0.04 | -0.07 | -0.09 | 0.10 | 0.12 |
| **GAF** | 0.08 | 0.20 | 0.22 | -0.05 | 0.13 | 0.07 | 0.06 | 0.03 | 1 | -0.12 | 0.05 | 0.03 | -0.09 | -0.04 | -0.02 |
| **CF** | 0.08 | 0.07 | -0.08 | 0.25 | -0.33 | -0.05 | -0.06 | 0.08 | -0.12 | 1 | 0.56 | 0.056 | -0.17 | 0.10 | -0.05 |
| **DSST** | -0.06 | -0.01 | 0.14 | 0.05 | -0.12 | 0.08 | 0.11 | 0.04 | 0.05 | 0.56 | 1 | 0.37 | 0.25 | -0.23 | -0.002 |
| **TMT** | 0.05 | 0.02 | -0.04 | 0.10 | 0.28 | 0.04 | -0.002 | -0.07 | 0.03 | 0.06 | 0.37 | 1 | 0.24 | -0.14 | 0.05 |
| **Age** | -0.04 | -0.23 | 0.12 | 0.05 | 0.14 | 0.07 | -0.05 | -0.09 | -0.08 | -0.17 | 0.25 | 0.24 | 1 | 0.23 | 0.02 |
| **Edu** | 0.04 | -0.07 | 0.01 | -0.05 | 0.04 | -0.14 | 0.06 | 0.10 | -0.04 | 0.09 | -0.23 | -0.14 | 0.23 | 1 | -0.03 |
| **Med** | -0.14 | 0.03 | 0.10 | -0.10 | 0.07 | -0.07 | 0.15 | 0.12 | -0.02 | -0.05 | -0.002 | 0.052 | 0.02 | -0.03 | 1 |

*Non-network* partial correlations between variables in our study sample (n=212) including four covariates: age, sex, education, medication.

Abbreviations: *r* partial correlation coefficient, *PANSS* Positive and Negative Symptoms Scale, *NSS* Neurological Soft Signs, *P* *PANSS* Positive, *N* *PANSS* Negative, *G* *PANSS* General, *MOCO* Motor Coordination, *SI* Sensory Integration, *RLSPO* Right/Left Spatial Orientation, *HS* Hard Signs, *GAF* Global Assessment of Functioning Scale, *CF* Category Fluency, *DSST* Digit Symbol Substitution Test, *TMT-B* Trail Making Test part B.

**Supplementary table 4.**

| ***p*** |  |  |  |  |  |  |  |  |  |  |  |  |  |  |  |
| --- | --- | --- | --- | --- | --- | --- | --- | --- | --- | --- | --- | --- | --- | --- | --- |
|  | **P** | **N** | **G** | **MOCO** | **SI** | **COMT** | **RLSPO** | **HS** | **GAF** | **CF** | **DSST** | **TMT** | **Age** | **Edu** | **Med** |
| **P** | 0 | 0.41 | **2.87e-15** | 0.17 | 0.96 | 0.11 | 0.21 | 0.72 | 0.25 | 0.25 | 0.38 | 0.48 | 0.55 | 0.54 | 0.05 |
| **N** | 0.41 | 0 | **2.00 e-11** | 0.65 | 0.05 | 0.66 | 0.55 | 0.78 | **0.01** | 0.32 | 0.88 | 0.80 | **0.001** | 0.34 | 0.64 |
| **G** | **2.87e-15** | **2.00e-11** | 0 | 0.88 | 0.47 | 0.89 | 0.08 | 0.52 | **0.002** | 0.26 | 0.05 | 0.54 | 0.10 | 0.84 | 0.14 |
| **MOCO** | 0.17 | 0.65 | 0.88 | 0 | **5.8e-05** | **0.0003** | **0.003** | 0.96 | 0.47 | **0.0003** | 0.52 | 0.15 | 0.45 | 0.45 | 0.18 |
| **SI** | 0.96 | 0.05 | 0.47 | **5.84e-05** | 0 | 0.46 | 0.08 | **3.0e-06** | 0.06 | **1.55e-06** | 0.10 | **6.17e-05** | 0.04 | 0.59 | 0.35 |
| **COMT** | 0.11 | 0.66 | 0.89 | **0.0003** | 0.46 | 0 | **0.003** | **0.003** | 0.29 | 0.50 | 0.23 | 0.54 | 0.33 | **0.04** | 0.34 |
| **RLSPO** | 0.21 | 0.55 | 0.08 | **0.003** | 0.08 | **0.003** | 0 | 0.16 | 0.38 | 0.38 | 0.13 | 0.98 | 0.45 | 0.42 | 0.04 |
| **HS** | 0.72 | 0.78 | 0.52 | 0.96 | **3.06e-06** | **0.003** | 0.16 | 0 | 0.68 | 0.24 | 0.55 | 0.34 | 0.20 | 0.17 | 0.10 |
| **GAF** | 0.25 | **0.01** | **0.002** | 0.48 | 0.06 | 0.29 | 0.38 | 0.68 | 0 | 0.08 | 0.47 | 0.71 | 0.21 | 0.62 | 0.82 |
| **CF** | 0.25 | 0.31 | 0.26 | **0.0003** | **1.55e-06** | 0.50 | 0.38 | 0.24 | 0.08 | 0 | 8.61e-18 | 0.423 | **0.01** | 0.17 | 0.45 |
| **DSST** | 0.38 | 0.88 | 0.05 | 0.52 | 0.10 | 0.23 | 0.13 | 0.55 | 0.47 | **8.61e-18** | 0 | **8.38e-08** | **0.0004** | **0.001** | 0.98 |
| **TMT** | 0.48 | 0.80 | 0.54 | 0.15 | **6.17e-05** | 0.54 | 0.98 | 0.34 | 0.71 | 0.43 | **8.38e-08** | 0 | **0.0005** | **0.04** | 0.47 |
| **Age** | 0.55 | **0.001** | 0.10 | 0.44 | 0.04 | 0.33 | 0.45 | 0.20 | 0.22 | **0.01** | **0.0003** | **0.0005** | 0 | **0.001** | 0.80 |
| **Edu** | 0.54 | 0.34 | 0.84 | 0.45 | 0.59 | **0.04** | 0.42 | 0.17 | 0.62 | 0.17 | **0.001** | **0.04** | **0.001** | 0 | 0.64 |
| **Med** | 0.05 | 0.64 | 0.14 | 0.18 | 0.35 | 0.34 | **0.04** | 0.10 | 0.82 | 0.45 | 0.98 | 0.47 | 0.80 | 0.64 | 0 |

*Non-network* partial correlations between variables in our study sample (n=212) including four covariates: age, sex, education, medication.

Abbreviations: *p* p-value uncorrected for multiple testing (significant values are marked in bold)*, PANSS* Positive and Negative Symptoms Scale, *NSS* Neurological Soft Signs, *P* *PANSS* Positive, *N* *PANSS* Negative, *G* *PANSS* General, *MOCO* Motor Coordination, *SI* Sensory Integration, *RLSPO* Right/Left Spatial Orientation, *HS* Hard Signs, *GAF* Global Assessment of Functioning Scale, *CF* Category Fluency, *DSST* Digit Symbol Substitution Test, *TMT-B* Trail Making Test part B.
